# Quality in Question: Assessing the Accuracy of Four Heart Rate Wearables and the Implications for Psychophysiological Research

**DOI:** 10.1111/psyp.70004

**Published:** 2025-02-04

**Authors:** Mohammadamin Sinichi, Martin J. Gevonden, Lydia Krabbendam

**Affiliations:** ^1^ Department of Clinical, Neuro‐ & Developmental Psychology, Faculty of Behavioural and Movement Sciences Vrije Universiteit Amsterdam Amsterdam The Netherlands; ^2^ Institute Brain and Behaviour (iBBA) Amsterdam The Netherlands; ^3^ Department of Biological Psychology, Faculty of Behavioural and Movement Sciences Vrije Universiteit Amsterdam Amsterdam The Netherlands

## Abstract

Heart rate (HR) and heart rate variability (HRV) are two key measures with significant relevance in psychophysiological studies, and their measurement has become more convenient due to advances in wearable technology. However, photoplethysmography (PPG)‐based wearables pose critical validity concerns. In this study, we validated four PPG wearables: three consumer‐grade devices (Kyto2935, Schone Rhythm 24, and HeartMath Inner Balance Bluetooth) and one research‐grade device (Empatica EmbracePlus, successor to the widely‐used but discontinued Empatica E4). All devices were worn simultaneously by 40 healthy participants who underwent conditions commonly used in laboratory research (seated rest, arithmetic task, recovery, slow‐paced breathing, a neuropsychological task, posture manipulation by standing up) and encountered in ambulatory‐like settings (slow walking and stationary biking), compared against a criterion electrocardiography device, the Vrije Universiteit Ambulatory Monitoring System (VU‐AMS). We determined the signal quality, the linear strength through regression analysis, the bias through Bland–Altman analysis, and the measurement error through mean arctangent absolute percentage error for each condition against the criterion device. We found that the research‐grade device did not outperform the consumer‐grade devices in laboratory conditions. It also showed low agreement with the ECG in ambulatory‐like conditions. In general, devices captured HR more accurately than HRV. Finally, conditions that deviated from baseline settings and involved slight to high movement, negatively impacted the agreement between PPG devices and the criterion. We conclude that PPG devices, even those advertised and designed for research purposes, may pose validity concerns for HRV measurement in conditions other than those similar to resting states.

## Impact statement

Our results contribute to the literature by urging caution in psychophysiological studies that employ consumer‐grade or research‐grade optical‐based sensors to measure heart rate and heart rate variability. We provide evidence that the accuracy of such devices deteriorates when subjects are in motion, by decreasing signal‐to‐noise ratio, and masking meaningful interpretations.

## 1 Introduction

### 1.1 Background

Heart rate (HR), defined as the number of times the heart beats per minute, has long been a cornerstone of cardiovascular psychophysiological research. In recent years, its prominence has been overshadowed by the rising popularity of heart rate variability (HRV), which is defined as the variation between successive beat‐to‐beat intervals (Malik et al. 1996; Shaffer, McCraty, and Zerr 2014). As reviewed by Shaffer, McCraty, and Zerr (2014) and Laborde, Mosley, and Thayer (2017), there are different theoretical frameworks attempting to explain the mechanisms through which HRV arises and why it covaries with psychological phenomena (Grossman and Taylor 2007; Thayer et al. 2009; Porges 2007; Lehrer and Gevirtz 2014; McCraty and Childre 2010; Laborde, Mosley, and Mertgen 2018). Accordingly, several studies have provided empirical evidence that resting‐state HRV is associated with an array of cognitive functions, including working memory (Hansen, Johnsen, and Thayer 2003; Zeng et al. 2023), executive functions (Williams et al. 2019), self‐regulation and inhibition (Williams et al. 2015; Mantantzis, Schlaghecken, and Maylor 2020; Ottaviani et al. 2019), decision‐making (Fung et al. 2017; Wegmann et al. 2020), and emotion regulation (Mantantzis, Schlaghecken, and Maylor 2020; Sakaki et al. 2016).

The gold standard method for HRV measurement is electrocardiography (ECG), which measures the electrical activity of the heart's muscles (Akselrod et al. 1981; Malik et al. 1996; Shaffer, McCraty, and Zerr 2014; Laborde, Mosley, and Thayer 2017; Quigley et al. 2024). The most pertinent cardiac aspect for analyzing HRV is the contraction of the ventricles, responsible for pumping blood to the organs and lungs, as represented by the QRS complex on the electrocardiogram. The distinct sharpness and amplitude of the R‐peak make it the landmark of choice for algorithms to identify and calculate the interbeat interval (IBI), representing the length of one cardiac cycle (also referred to as a heart period, but we will stick to IBI throughout). These IBIs can then be used to calculate various HRV outcome indices across different domains: the time domain (e.g., pvRSA, SDNN, PNN50, and RMSSD), the frequency domain (e.g., high‐frequency and low‐frequency power), and nonlinear measures (e.g., SD1 and SD2). The metrics differ in their sensitivity to artifacts, suitability for various epoch sizes, physiological interpretation, applicability to certain populations, and responsiveness to interventions (for a comprehensive guide, see Shaffer and Ginsberg 2017). Therefore, some metrics are valued for very specific use cases in research and clinical practice.

Among these HRV metrics, the root mean squared of successive differences (RMSSD) and the high‐frequency component (HF) are two of the most generally used measures in psychophysiological research (Laborde, Mosley, and Thayer 2017; Laborde et al. 2023; Shaffer, McCraty, and Zerr 2014; Shaffer and Ginsberg 2017). This is because both are highly correlated with respiratory sinus arrhythmia, which is an index of cardiac control tied to the respiratory cycle, and considered to be primarily modulated through the vagus nerve (Pomeranz et al. 1985). Consequently, RMSSD and HF‐HRV are also highly correlated with one another. Empirical studies have shown that these measures of variability are reduced after parasympathetic blockage (Akselrod et al. 1981; Penttilä et al. 2001; Pomeranz et al. 1985), suggesting that they, to some extent, provide a proxy for the parasympathetic branch of the autonomic nervous system. HF‐HRV reflects the power within a predefined frequency band linked to respiration (between 7.2 and 24 breaths per minute, corresponding to a 0.12–0.40 Hz frequency band), whereas RMSSD has been shown to be relatively less specific to respiration and include more variability from other sources (Hill and Siebenbrock 2009; Penttilä et al. 2001; Quigley et al. 2024). However, RMSSD does retain high‐pass filter properties that allow it to capture the rapid, phasic modulation of the vagus nerve over the heart's sinoatrial node (Berntson, Lozano, and Chen 2005).

While ECG is accurate, its reliance on patches and wired connections with wet electrodes on the thorax poses practical limitations, especially for extended monitoring periods in ambulatory settings. While this may be acceptable to patient populations motivated by potential clinical gain, it is a much harder sell to general research participants. Wet electrodes, in particular, are associated with additional time and attention investment due to the need for frequent reapplication, and long‐term use can lead to skin irritation. To a lesser extent, this also applies to dry electrode ECG systems, which typically rely on chest strap sensors. Both ECG varieties face further challenges, including the need to recharge due to limited battery life and the need to offload high‐frequency ECG data due to limited device memory. Consequently, there is growing interest in using devices that employ optical sensors to measure cardiac activity through photoplethysmography (PPG). Devices equipped with this technique detect variations in blood volume in a particular region, such as the earlobe, wrist, or finger, which are thought to correspond with the heart's contractions (Challoner and Ramsay 1974), albeit with a slight lag in the blood's arrival to the tissue (Jago and Murray 1988). The maximum blood volume in the PPG waveform is detected as the systolic peak, allowing for calculations of IBIs.

Such PPG devices can be categorized as research‐grade devices (designed primarily for clinical or research purposes) and consumer‐grade devices (designed for use by health‐conscious consumers for self‐monitoring or biofeedback). Although the boundary is somewhat arbitrary, research‐grade devices are primarily marketed to professional clients, typically offer access to signal‐level data, and are presented as adhering to more rigorous scientific standards, prioritizing validity and reliability. Importantly, the term “research‐grade” is a self‐designation used by these wearables. It does not imply adherence to data quality or research validation standards set by any regulation, as no such standards currently exist. In contrast, consumer‐grade devices are widely available through online marketplaces and are designed for a general audience, prioritizing end‐user experience, affordability, and ease of use, with greater emphasis on feature‐level data presented in dashboard form. Due to their low cost, and low user burden designs, the latter are also widely employed in research (Bent et al. 2020). The PPG wearable devices also have different use cases: Some are primarily designed to be used in a stationary setting to measure resting‐state HRV, deliver biofeedback, and measure during stress‐inducing or cognitively demanding tasks, while others are designed for longer term naturalistic monitoring in conditions that involve components similar to a person's everyday life functioning, including dynamic activities such as walking, biking, and exercise.

Despite its practical benefits, PPG poses accuracy limitations. One such limitation is that the waveform of PPG is more blunted than the pronounced, high‐amplitude R peaks observed in ECG. Consequently, this characteristic complicates the task for algorithms in identifying the systolic peak and increases susceptibility to movement artifacts (Allen 2007; Schäfer and Vagedes 2013). Furthermore, considering the operational mechanisms of PPG‐based wearables, which rely on detecting light reflected from blood vessels, these devices tend to perform suboptimally when there is a higher concentration of melanin in the skin, particularly in cases of darker skin tones (Hill et al. 2015; Shcherbina et al. 2017). Subsequently, using PPG wearables to quantify HR and HRV, especially in nonlaboratory settings, typically means a trade‐off between increased acceptability by participants at the cost of reduced validity and reliability of the measures. These challenges are not limited to consumer‐grade devices but also affect research‐grade devices. Hence, as suggested by many recent reviews (Alugubelli, Abuissa, and Roka 2022; Charlton et al. 2023; Cosoli, Antognoli, and Scalise 2023; D'Angelo et al. 2023; Quigley et al. 2024), it is crucial to properly validate PPG‐based heart rate sensors before using them in research. Importantly, validation studies should cover a variety of use cases so that researchers can select the devices that will yield the data quality necessary to answer the research question at hand. This study aimed to do this by evaluating an array of PPG devices that have recently entered the market with potential for research use in laboratory and ambulatory settings but lack external validation.

Numerous studies have demonstrated the challenges associated with using PPG devices in experimental designs that involve participant movement. For instance, Bent et al. (2020) investigated the accuracy of PPG‐based HR in the main smart watches and wristbands available on the market at the time (Apple Watch, Fitbit, Garmin, Xiaomi, Biovotion, Empatica E4) and reported that the absolute error during activity was on average 30% higher than during rest. Another study by Lindsey et al. (2023) assessed several consumer‐grade PPG devices and indicated that the accuracy of wrist‐worn PPG monitors is dependent on the level of physical activity, and tend to underestimate HR at lower exercise intensities and overestimate it during intense workouts. Similarly, a study by Wang et al. (2017), which tested the FitBit, Apple Watch, Mio Alpha, and Basis Peak, concluded that their accuracy diminished with increased activity.

This issue becomes more problematic when calculating HRV indices, which rely on precise detection of systolic peaks. In another study, Van Voorhees et al. (2022) compared the research‐grade Empatica E4 to a Holter ECG in an ambulatory design for HRV measurement and revealed low reliability, questioning its use in settings where participant movement is not under experimental control. Similarly, Menghini et al. (2019) tested the Empatica E4 in various conditions, including laboratory and ambulatory settings. They found that in conditions involving movement (e.g., walking), the accuracy of the measured HRV indices significantly diminished. Hoog Antink et al. (2021) also used a wrist‐worn PPG device in ambulatory settings and concluded that HRV outcome measures, especially those influenced by high frequency, should be used with caution due to their susceptibility to larger errors. The latter result was recently replicated by Hu et al. (2024), who demonstrated that HRV indices collected with the Empatica E4 exhibit lower agreement with ECG measurements compared with the agreement for HR. Additionally, hand movement was negatively correlated with the signal's valid data rate, and the valid data rate was lower in ambulatory settings than in laboratory conditions. These findings, along with a substantial body of similar research (Castaneda et al. 2018; Dobbs et al. 2019; Georgiou et al. 2018; Lu et al. 2009; Nelson et al. 2020; Peake, Kerr, and Sullivan 2018; Schäfer and Vagedes 2013), highlight the fact that PPG wearables tend to have reduced accuracy when the subject is in motion. Since devices are often marketed for exercise use, this may lead to a mismatch between device performance and researcher expectations.

### 1.2 The Current Study

In this study, we have validated four recently released PPG wearables with potential for use in psychophysiological studies. Three are consumer‐grade: Kyto2935, Schone Rhythm 24, and HeartMath Inner Balance Bluetooth; and one is research‐grade, the Empatica EmbracePlus. Empatica EmbracePlus is the successor of the Empatica E4, whose performance has been evaluated in previous studies (Bent et al. 2020; Hu et al. 2024; Menghini et al. 2019; Van Voorhees et al. 2022). To our knowledge, there are no external validation studies on the wearable devices currently selected for this study; however, they are marketed with the potential to be applied in diverse research settings: Kyto and HeartMath, primarily for resting‐state HRV measurement and biofeedback; Rhythm, when set to HRV mode, for resting‐state HRV measurement; and Empatica, for both laboratory experiments and ambulatory monitoring. These wearables cover a broad spectrum of features, enabling us to compare their diverse attributes. The devices differ in their placement: The Empatica is worn on the wrist, the Rhythm on the arm, and the HeartMath and Kyto use an ear clip attached to the earlobe. The quality of their sensors also varies in aspects such as channel configurations, wavelength capabilities, and sampling rates: Empatica samples at 64 Hz, HeartMath and Rhythm at 125 Hz, and Kyto at 1024 Hz.

The rationale behind selecting these particular wearables lies in their common advantages: (a) All selected devices transfer IBIs, which are essential for HRV analysis. While most consumer‐grade devices on the market only offer aggregated HR or HRV values (e.g., averages over a minute) or require a paid SDK to access higher resolution data, the devices chosen in this study by default can transmit a time series of detected IBIs via Bluetooth. Empatica stores systolic peaks detected by its proprietary algorithm on‐board of the device, which then allows for the derivation of IBI time series. Empatica also offers raw PPG signals, but this study focuses on the validation of the IBI‐level data. (b) All devices are easy to use: Data collection with these devices can be conducted by research assistants with minimal training and without requiring extensive additional equipment. All devices can be synchronized and initiated via a smartphone or tablet. (c) Relatively low cost and high accessibility: The consumer‐grade devices in this study are low‐cost and readily available through online marketplaces. The research‐grade device is also easily purchased online, and the large body of publications with its discontinued predecessor, Empatica E4, demonstrate that its higher price point is still acceptable for many researchers. These features make the chosen devices potential candidates for HR and HRV monitoring; they provide some form of relatively low‐level data, are scalable to some extent, and have potential applications in diverse psychophysiological contexts. Consequently, they may be considered viable options for researchers aiming to monitor HR and HRV in various settings.

In a 90‐min study with 40 participants, we employed eight experimental conditions that mimic both laboratory and ambulatory settings to test the accuracy of these wearables in measuring HR and HRV, as compared to a criterion ECG device, the Vrije Universiteit Ambulatory Monitoring System (VU‐AMS 5 fs) (de Geus et al. 1995; Willemsen et al. 1996). These conditions include measurement conditions for which these wearables are designed and marketed: laboratory‐like settings with no movement for resting‐state HRV measurement, cognitive task conditions common in psychophysiological testing, slow‐paced breathing, stress induction, posture manipulation, and ambulatory‐like settings involving slow‐paced walking and stationary biking. We tested all devices under each condition, although only Empatica is specifically designed for ambulatory settings.

Through signal quality assessment, mean arctangent absolute percentage error (MAAPE), regression analysis, and Bland–Altman analysis, we evaluated the agreement of each device under each condition with the criterion device (higher agreement was characterized by higher signal quality, lower MAAPE, higher correlation coefficients, and lower Bland–Altman biases). We hypothesized that: (1) All PPG devices would show higher agreement with the criterion ECG (VU‐AMS) when measuring HR compared to HRV (RMSSD, HF‐HRV). (2) All PPG devices would show higher agreement with the criterion in laboratory‐like conditions compared to ambulatory‐like conditions, for both HR and HRV. (3) The new Empatica EmbracePlus, marketed as a research‐grade device, would perform better (i.e., show higher agreement) than consumer‐grade devices (Kyto, Rhythm, and HeartMath) in laboratory‐like conditions. (4) Empatica would show high agreement with the criterion in ambulatory‐like conditions (given its wrist‐band design and marketing for daily life monitoring use‐cases), whereas the other consumer‐grade devices would show relatively lower agreement. The analyses were conducted using a self‐developed open‐source Python pipeline (Wearable HRV, Sinichi, Gevonden, and Krabbendam 2024), which facilitates reproducibility and sets the stage for future validation studies. Finally, we share several insights regarding the use of PPG wearables in psychophysiological research for measuring HR and HRV.

## 2 Method

### 2.1 Participants

Forty‐one participants were recruited through various methods, including the Faculty of Behavioural and Movement Sciences (FGB) Subject Pool system (Sona) at Vrije Universiteit Amsterdam, distributing flyers at the university and on social media, and through personal networks. Participation was compensated with either research credits (for students) or 17 euros. One participant was excluded due to a high frequency of artifacts and ectopic beats. The final sample size was 40 participants (29 female), with an average age of 24.23 years (SD = 6.52 years), a mean Body Mass Index (BMI) of 23.66 kg/m^2^ (SD = 3.70), average height 170.88 cm (SD = 9.62), and average weight 69.48 kg (SD = 14.54). 90% were right‐handed and 10% left‐handed. Participants were categorized by skin tone using Von Luschan's chromatic scale as follows: 30.0% light or light‐skinned European, 52.5% light intermediate or dark‐skinned European, and 17.5% dark intermediate or olive skin, with no dark/brown type or very dark/black type participants. Six of the participants did not wear the Empatica EmbracePlus because it was incorporated at a slightly later stage in the experiment. The number of participants included in the final analyses varied due to issues such as device malfunction and connectivity problems (summarized in Table S1). The Local Ethics Committee of the Faculty of Behavioural and Movement Sciences of Vrije Universiteit Amsterdam (in Dutch: Vaste Commissie Wetenschap en Ethiek, VCWE) approved this study (reference number: VCWE‐2023‐041). Informed consent was obtained from all participants.

### 2.2 Exclusion Criteria and Controlling for Confounders

All participants received an information letter before the study, which included inclusion and exclusion criteria. In accordance with general protocols in psychophysiological studies (Laborde, Mosley, and Thayer 2017; van der Mee et al. 2021; Ziemssen and Siepmann 2019), participants could not participate in the study if they had serious diseases affecting major organs (heart, lungs, liver, and kidneys) or conditions causing malignancy or hematological disorders. Those with known diabetes, diagnosed neuropathy, or on medications affecting cardiac activity were also excluded. Metabolic disorders such as uncontrolled thyroid or adrenal diseases, alcohol abuse (over 21 units per week for men and over 14 for women), pregnancy or breastfeeding, an inability to understand the study protocol due to language barriers, an inability to give informed consent, or a disability (visual, reading, or physical) preventing participation in all study conditions were additional exclusion criteria. Furthermore, individuals with infectious earlobes or nonremovable earrings were ineligible, given the placement of two heart rate monitors on the earlobes. Prior to the study, participants were instructed to adhere to several guidelines: Maintaining a normal sleep schedule the night before the experiment, avoiding intense physical activity the day before, refraining from eating in the last two hours before the experiment, avoiding consumption of coffee or caffeinated drinks in the two hours prior to the experiment, and abstaining from alcohol for 24 h before the experiment.

### 2.3 Power Analysis

Our study is well‐powered to conduct group statistical analyses, even with data loss due to the malfunctioning of some devices (discussed later in Signal Quality section). A priori power analysis using the G*Power software (version 3.1.9.7) (Faul et al. 2007) indicated that for regression analysis to detect large effect sizes, based on Cohen's suggestion ($f^{2}$ = 0.35), with an alpha error of 0.05 and an expected power of 0.80 with one predictor, 20 participants are sufficient.

### 2.4 Recording Devices

We used the 5 fs version of the Vrije Universiteit Ambulatory Monitoring System (VU‐AMS), as the criterion device (de Geus et al. 1995; Willemsen et al. 1996), which has been used extensively in research as a validated and reliable tool for continuous ECG monitoring in both laboratory and ambulatory settings (de Geus and van Doornen 1996; van der Mee et al. 2021). The VU‐AMS 5 fs samples ECG at a rate of 1000 Hz with a 16‐bit resolution. During the experiment, the device was worn on the left hip using a belt. Three AgCl surface electrodes (Kendall H98SG, Cardinal Health) were positioned on the chest at the suprasternal notch, the left lateral margin near the apex of the heart (ground electrode), and the processus xiphodius (for ECG); two on the back, aligned with the spine slightly above and below the level of the chest electrodes (for impedance cardiography—not analyzed in this experiment).

**FIGURE 1:**
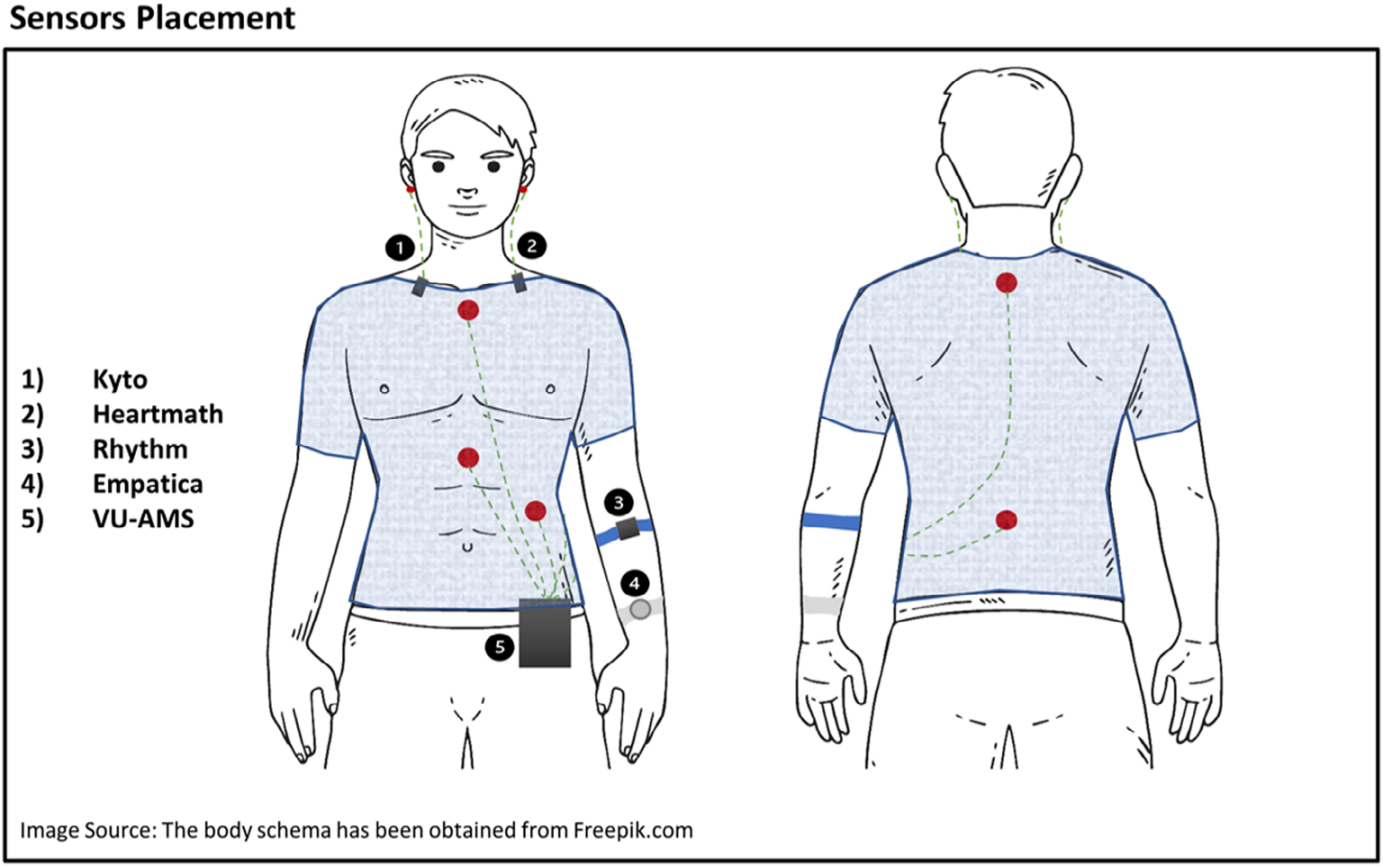
Sensor Placement: The figure illustrates the placement of each recording device. Two devices were placed on the earlobe (Kyto and HeartMath), one on the upper arm (Rhythm), one on the wrist (Empatica), and the criterion device (VU‐AMS) on both the chest and the back.

We selected four PPG heart rate devices to compare against the criterion device: Kyto2935, Schone Rhythm 24, HeartMath Inner Balance Bluetooth sensor, and Empatica EmbracePlus. All these devices are capable of detecting and transmitting IBIs, have the potential to be used in research, and offer versatility in data collection. For simplicity, we will refer to these devices as Kyto, Rhythm, HeartMath, and Empatica. Figure 1 illustrates the placement of each device, and the layout for the placement of the ECG electrodes.

Kyto (by KYTO Fitness Technology Co. Ltd.) is designed to be worn on the earlobe and features a PPG sampling rate of 1024 Hz with one green LED light. HeartMath (by HeartMath Inc) is worn on the other earlobe and measures PPG at a 125 Hz sampling rate, also featuring one green LED light. Rhythm (by Scosche Industries Inc.) is worn on the arm with a sampling rate of 125 Hz. We set the recording mode to “heart rate variability,” the only mode that allows for the export of beat‐to‐beat data. Rhythm features two green and one yellow LED light. The Empatica EmbracePlus (by Empatica Inc.) has a PPG sensor with a sampling rate of 64 Hz, featuring four acquisition channels (via eight photodiodes) and nine LEDs with wavelengths of Red, Infra‐Red, and Green.

Kyto, Rhythm, and HeartMath do not store or transmit raw PPG data but have a Bluetooth service for transmitting IBIs. To store the time series for detected IBIs, the devices were each connected via Bluetooth to an Android application, HRV Logger (Plews et al. 2017), on three dedicated phones (Redmi Note 10S). The IBIs detected by these devices were transferred to the app, along with UNIX time stamps (in milliseconds), and could then be saved as a .csv file on the phone. Empatica was paired through Bluetooth with its companion app, “CareLab,” which transfers the recorded data to a cloud storage environment, from which the raw data, and the detected systolic peaks could be retrieved. To align with the analyses for the other three devices, this study solely validates the IBIs detected through the device's proprietary peak detection algorithm, not the quality of the underlying PPG signal.

### 2.5 Procedure

The study procedure took approximately 90 min to complete. Upon arrival at the laboratory, the experiment's goals were explained, and participants had the opportunity to review the information letter, previously received via email. After signing informed consent, their height and weight were measured. The VU‐AMS electrodes were then attached using the standard configuration and secured to a belt. Empatica was placed on the wrist of the non‐dominant hand, one finger's distance from the pisiform bone, and connected to the “CareLab” Android application on a dedicated phone. Participants completed several demographic questionnaires on Qualtrics (Qualtrics, Provo, UT), while resting quietly, which approximately took 15 min to complete. The details of these questionnaires are outside the scope of this study as they were intended to answer an unrelated research question, but this period effectively served as a habituation period to the environment and to the ECG device, before starting the recordings.

After completing the questionnaires, the remaining sensors were placed. Kyto and HeartMath were attached to the earlobe, alternating between the right or left ear for different participants. The wires were taped on the neck with medical tape to prevent detachment or movement artifacts. The Rhythm manual suggests placing the sensor on the upper arm (recommended), biceps, and triceps. We always started by placing it on the nondominant hand's upper arm; however, for some participants, no IBIs were detected at that location. In those cases, alternative locations such as the biceps, triceps, and also the upper arm of the dominant hand were tried until the device started transmitting IBIs. The VU‐AMS device was then connected to the VU‐DAMS software (version 5.4.20, available at http://www.vu‐ams.nl/vu‐ams/software/) on a laptop (Surface Laptop 4) via Bluetooth, and signal quality was checked. Electrodes were replaced if necessary to achieve acceptable signal quality. Recording on the VU‐AMS device and the other four wearables then began simultaneously. Participants performed a series of tasks in a fixed order: sitting, arithmetic task, recovery phase, standing, slow‐paced breathing, emotional go/no‐go task, slow‐walking, and stationary biking. After completing all conditions, the sensors and electrodes were removed, and participants were debriefed.

#### 2.5.1 Experimental Conditions

The experimental protocol consists of eight conditions. Each condition lasted for 3 min, with the exceptions of the seated rest, which lasted for 5 min, and the emotional go/no‐go task, which took approximately 10 min. These conditions encompass both laboratory‐ and ambulatory‐like situations commonly used in psychophysiology research. These conditions were chosen to induce changes in HR and HRV and differ in PPG signal quality.

##### 2.5.1.1 Seated Rest

The participants were instructed to adopt a stable sitting posture, with their knees bent at a 90° angle, feet firmly placed on the floor, hands resting on their thighs with palms facing upwards, eyes closed, and they were instructed to breathe normally and spontaneously (Laborde, Mosley, and Thayer 2017).

##### 2.5.1.2 Arithmetic Task

Participants were instructed to perform a mental subtraction task. They were asked to subtract the Number 13 from 1022 and verbally report their answers out loud. If they made a mistake or forgot a number, they were asked to start again from the initial number. The experimenter did not record the participants' answers. Participants were instructed to keep their hands on their thighs and not to move. In case of movement, they were reminded to sit as still as possible. The nature of the task makes it mentally challenging, and the attitude of the experimenter adds a social evaluation component to it. Moreover, it contains other components such as speech, and potential muscle contraction, that can all change HR and HRV and affect the signal quality.

#### 2.5.2 Recovery Phase

After the mental arithmetic task, participants were asked to sit still without moving or speaking for another three minutes. The position was the same as seated rest, except that participants could keep their eyes open.

#### 2.5.3 Standing Posture

Participants were asked to stand up without any body movement, and hang their hands along their sides.

#### 2.5.4 Slow‐Paced Breathing

In order to increase the HRV (Laborde et al. 2022; Lehrer and Gevirtz 2014; Sevoz‐Couche and Laborde 2022), participants remained seated and were instructed to synchronize their breathing with a breathing pacer on the screen set to six breaths per minute, inhaling through the nose (4 s), and exhaling through the mouth (6 s).

#### 2.5.5 Neuropsychological Task

The integration of neuropsychological tasks is a standard practice in laboratory‐based psychophysiological assessments. We utilized an emotional go/no‐go task (Tottenham, Hare, and Casey 2011), implemented on INQUISIT platform (https://www.millisecond.com/). The task involved participants viewing images of facial expressions representing different emotions (fearful, happy, sad, angry, or neutral) and pressing the spacebar in response to specific expressions as instructed. There was a total of eight conditions in the task. Half of these conditions required participants to press the spacebar for a specific emotion (“go” for emotional faces, and “no‐go” for neutral faces), while the other half required them to press the spacebar for neutral faces (“go” for neutral faces, “no‐go” for emotional faces). Each condition consisted of 30 trials, in a randomized order of 20 “go” trials and 10 “no‐go” trials. The trial sequence began with the presentation of an image for 500 ms, followed by a fixation cross for 1000 ms, and a response timeout of 1500 ms. Ten adult faces (five females and five males) per facial expression were used from the NimStim database (Tottenham et al. 2009). We measured the face‐to‐screen distance and kept it at 60 cm for all participants. Participants were instructed not to move, and to only use the hand that had no heart rate sensors on it to press the response button on the keyboard.

#### 2.5.6 Slow Walking

Participants were asked to walk at a comfortable pace back and forth around the laboratory, allowing their hands to move naturally as they walked. The researcher initially demonstrated an example for the participants to follow.

#### 2.5.7 Stationary Biking

Participants were asked to ride a stationary bike, maintaining a pedal speed of 30 rpm (RPM) for the duration of the measurement, with the resistance set to 120 W. They were instructed to hold the bike hand grips, keeping their hands steady without squeezing their muscles.

For simplicity, these conditions will be referred to as follows throughout the manuscript: sitting, arithmetic, recovery, standing, breathing, neurotask, walking, and biking.

### 2.6 Data Processing

This section outlines the data processing for each device, which due to differences in data formats, required slightly different processing steps. For VU‐AMS ECG data, peaks were detected and cleaned using the VU‐DAMS software. IBIs detected by the internal device algorithms of Kyto, Rhythm, and HeartMath were exported via Bluetooth to the HRV Logger app. Similarly, Empatica's detected systolic peaks were transferred to an online server, and then extracted and converted to IBIs. All data were synchronized to a unified time format and pre‐processed using the “WearableHRV” Python package. This included Karlsson artifact correction and the removal of physiologically implausible data with linear interpolation. The preprocessed data were then segmented according to experimental conditions, and mean HR, RMSSD, and HF‐HRV metrics were extracted for each condition and device. Finally, we assessed the quality of the signal for each device and condition by setting thresholds for signal‐to‐noise ratio and the number of IBIs compared to the criterion. Additionally, wrist acceleration was calculated based on data from Empatica's accelerometer. The detail for each step is explained below.

#### 2.6.1 Data Preparation

The .5FS VU‐AMS data were opened using the VU‐DAMS software and all R‐peaks were automatically detected and scored. The software flags potentially deviant or problematic peaks and allows to cycle through them starting with the most deviant ones. These beats were visually inspected within their surrounding 10‐s time window and if necessary, the R‐peaks were manually adjusted. The IBIs, along with their corresponding timestamps, were then exported as a .txt file.

The detected IBIs by Kyto, Rhythm, and HeartMath were transferred to HRV Logger and saved together with time stamps as a .csv file. For Empatica, we used a customized Python script to open the .avro files saved on the cloud storage, each of which contains approximately 15 min of data. This script collated these files and then extracted the automatically detected systolic peaks, along with their timestamps. Finally, we calculated the IBIs by taking the difference between two adjacent detected systolic beats and saved this data as a .csv file.

#### 2.6.2 Preprocessing

We used our self‐developed Python package, “WearableHRV,” as the validation pipeline (Sinichi, Gevonden, and Krabbendam 2024) For each participant, after data preparation, all files containing IBIs and timestamps were imported to the package in a Jupyter Notebook environment, run through Anaconda Navigator 3 (version 2.5.1). The time stamps from all devices were then converted to a mutual format (hh:mm:ss.ms). Subsequently, the time series from all devices were synchronized by maximizing the cross‐correlation through visual inspection and manual adjustments. The synchronized continuous data was segmented into intervals representing the experimental conditions.

Our pipeline employs another Python package, “hrvanalysis,” for outlier and ectopic beats preprocessing and data extraction (Champseix, Ribiere, and Couedic 2021). The segmented data for each condition, for each device, was independently run through the following steps: First, IBIs shorter than 300 ms or longer than 2000 ms were removed from the segments and were linearly interpolated. Then, to further identify and isolate the outliers, we adopted a personalized approach by employing the Karlsson method (Karlsson et al. 2012). We initially set a custom removal threshold of 0.25 (Lippman, Stein, and Lerman 1994). For each IBI (except the first and last), we calculated the mean of its preceding and succeeding IBIs. Each IBI was then compared with this mean. If the absolute difference between the current IBI and the mean exceeded 0.25 (the threshold) of this mean, the IBI was flagged as an outlier, removed, and linearly interpolated. Both types of outliers that were either detected with the 300–2000 threshold or with the Karlsson method are referred to as artifacts throughout this manuscript.

This approach enabled us to optimize the custom removal threshold for different individuals to avoid overcorrecting. Given that the raw ECG signal from the criterion device (VU‐AMS) underwent a visual inspection and subsequent preprocessing with the VU‐DAMS software, it was already clean and free of artifacts. Therefore, our goal was to adjust the custom removing threshold to avoid identifying outliers or ectopic beats in the criterion time series, while still being able to detect them in other devices. This consideration is essential because some individuals naturally exhibit higher heart rate variability; therefore, setting a threshold of 0.25 may incorrectly identify many of their IBIs as outliers, despite contrary indications from the raw ECG data. In such cases, we incrementally increased the custom removal threshold in steps of 0.05 until, for a given individual, we observed no further detection of outliers for the criterion device (see Figures S1 and S2 for additional info). Finally, the number of detected IBIs in each device for each condition, and the number of detected artifacts after pre‐processing were determined and stored.

#### 2.6.3 Feature Extraction

After preprocessing, using the integrated functionalities of the “hrvanalysis” Python package, we calculated various time domain and frequency domain metrics for HR and HRV. However, due to the number of devices used in our study for validation and to maintain report simplicity, we focused solely on three features: heart rate, and for HRV, the two most commonly used metrics, one from the time domain (root mean square of successive differences) and one from the frequency domain (high‐frequency component).

##### 2.6.3.1 Heart Rate (HR)

The heart rate was calculated as the number of heart beats per minute. This was determined by taking the average of the IBIs, and converting this average to beats per minute. The following formula was used:

$$\mathrm{HR}=\frac{60\;000}{\text{mⅇan}\left(\text{IBIs}\right)}$$

##### 2.6.3.2 Root Mean Square of the Successive Differences (RMSSD)

RMSSD was calculated as the square root of the mean of the sum of the squares of differences between adjacent IBIs. The formula for RMSSD is:

$$\text{RMSSD}=\sqrt{\frac{1}{N-1}\sum_{i=1}^{N-1}{\left({\mathrm{IBI}}_{i}-{\mathrm{IBI}}_{i-1}\right)}^{2}}$$

where ${\mathrm{IBI}}_{i}$ represents the *i*th inter‐beat interval, and N is the total number of IBIs.

##### 2.6.3.3 High Frequency (HF)

The HF component was calculated using Welch's method, with the IBIs interpolated to a regular time grid at a sampling frequency of 4 Hz to compute the Power Spectral Density (PSD). In accordance with the new guidelines for human HRV research (Quigley et al. 2024), the HF band, predefined in the range of 0.12 to 0.40 Hz, was then isolated from the PSD. The power within this HF band was integrated using the trapezoidal rule to obtain the total HF power. Since the HF band is tied to respiration, we confirmed that in 95% of cases, participants' respiration rates fell within the range of 7.2–24 breaths per minute. Deviations were primarily observed during conditions involving slow‐paced breathing, biking, walking, and neurotask. When respiration falls outside this range, it compromises the validity of HF‐HRV as a proxy for the parasympathetic arm. However, since the same band was applied consistently across all devices, this does not pose a threat to the interpretation of this study's results, as the comparison was made within individuals. The results for the HF measure are only reported in the Appendix S1.

#### 2.6.4 Signal Quality

After running the individual pipeline for each participant, the extracted features, number of detected IBIs, and number of artifacts (including outliers and ectopic beats) for each device and condition were imported into the group pipeline. As a first step, we assessed the signal quality by comparing data from each participant, device, and condition against the criterion device. We set a threshold of 30% for these comparisons and flagged a data segment as “poor signal” quality if: (a) the number of detected IBIs in that segment deviated by more than 30% from the criterion device (e.g., for one participant, the criterion detected 100 beats in a given condition, while one of the devices found only 60 beats for the same participant and condition), and (b) the number of detected artifacts in the segment exceeded 30% of the detected IBIs in that segment (e.g., 35 artifacts out of 100 detected IBIs).

During data collection, there were numerous instances where devices, especially Kyto and Rhythm, encountered issues. These issues included: (a) connectivity problems, where we were unable to connect a device to the host phone from the beginning; (b) the device disconnecting from the host phone during the experiment; (c) the inability to obtain a signal when placing the device in a given location; (d) Inability to retrieve the recorded data. In all instances where no data were recorded, either completely or in a given condition, we flagged them as “missing.” Importantly, we included the Empatica device from our seventh participant onwards. Therefore, the total number of participants for Kyto, Rhythm, and HeartMath is *N* = 40, and for Empatica, it is *N* = 34.

#### 2.6.5 Acceleration

We analyzed the wrist acceleration recorded via Empatica. These data were first converted from Analog to Digital converter (ADC) format into physical units (g) by calculating the sensitivity using the device's calibration parameters, which were computed as the ratio of the physical range to the digital range. Subsequently, a high‐pass filter with a cutoff frequency of 0.1 Hz and an order of 1 was applied to the *x*, *y*, and *z* components of the acceleration data to remove the static gravitational component. After filtering, the resultant acceleration magnitude was calculated using the Euclidean norm formula. The continuous time series data were then segmented based on predefined experimental conditions, and the mean acceleration magnitude over each condition was computed for each participant.

### 2.7 Data Analysis

We conducted three sets of statistical analyses to evaluate the validity of each of the PPG devices (Kyto, Rhythm, HeartMath, Empatica) against the criterion ECG device (VU‐AMS) within each condition. These include Mean Arctangent Absolute Percentage Error, regression analysis, and Bland–Altman analysis. In all analyses, comparisons are made between pairs of matching extracted features (RMSSD, HR, HF) for each condition, comparing each device against the criterion device. For regression, and Bland–Altman analysis, RMSSD and HF values were log‐transformed to adhere to a normal distribution. The correlation coefficient's interpretation ranges from “very high” for values between 0.90 and 1.00, “high” for 0.70 to 0.90, “moderate” for 0.50 to 0.70, “low” for 0.30 to 0.50, to “negligible” for values between 0 to 0.30 (Mukaka 2012). For the Bland–Altman and Mean Arctangent Absolute Percentage Error, fixed a priori benchmarks are not set; instead, relative comparisons between devices, or the same device under different conditions, will guide the interpretation of agreement levels.

#### 2.7.1 Mean Arctangent Absolute Percentage Error (MAAPE)

A common approach in the validation of wearables is using the Mean Absolute Percentage Error (MAPE). This calculation involves determining the absolute difference between each device's value and the criterion value (for each feature), normalized by the criterion value, and expressed as a percentage. However, specifically, for RMSSD values where there can be overestimation or underestimation by one device compared to the criterion, MAPE results in disproportionately high errors, distorting the scale of measurement, and causing interpretation issues. To address this, we have used a modified version of MAPE, namely the Mean Arctangent Absolute Percentage Error (MAAPE), which mitigates this issue (Kim and Kim 2016). Instead of using the percentage error directly as in MAPE, the arctangent function was applied to these errors. We then took the mean of these arctangent‐transformed percentage errors to quantify the average deviation of each device's measurements from the criterion. The following formula was used:

$$\text{MAAPE}=\frac{1}{N}\sum_{t=1}^{N}\arctan\left(\frac{\left|{\text{device}}_{t}-{\text{criterion}}_{t}\right|}{\left|{\text{criterion}}_{t}\right|}\right)$$

where MAAPE is the Mean Arctangent Absolute Percentage Error, N is the total number of observations, ${\text{criterion}}_{t}$ is the value from the criterion device at observation t, ${\text{device}}_{t}$ is the value from the device being tested at observation t, and arctan denotes the arctangent function.

#### 2.7.2 Regression Analysis

We utilized the “linregress” function from the “scipy.stats” module (Virtanen et al. 2020) to compute regression parameters. For each matching pair, we computed the slope, intercept, Pearson's correlation coefficient (*r*‐value), *p*‐value, standard error of the regression, and the number of cases included in the analysis.

#### 2.7.3 Bland–Altman Analysis

Pearson's regression only reflects the linear strength between data points, which is insufficient to establish the validity of the wearables (Bruton, Conway, and Holgate 2000). To address this, we further conducted a Bland–Altman analysis (Altman and Bland 1983). The Bland–Altman analysis involved calculating the mean difference (bias) of these differences for each matching pair. The calculation of bias and difference between the device and the criterion is done by subtracting the criterion measurements from the device measurements. Additionally, we computed the limits of agreement (LoA), defined as the bias ±1.96 times the standard deviation. This provides a range within which most differences between the device and the criterion measurements are expected to lie. 95% confidence intervals for bias and LoAs were also calculated.

### 2.8 Use of AI‐Generated Content (AIGC) and Tools

To enhance the clarity of sentences, improve grammar and syntax, and speed up programming, we made limited use of GPT‐4 (https://chat.openai.com/). Importantly, none of the content was generated purely by AI. All output from GPT‐4 was thoroughly and carefully reviewed before use. In cases where AI‐generated code was incorporated into an analysis, the outcomes of that analysis were carefully cross‐checked with analogous analyses in other statistical packages to ensure reliability.

### 2.9 Data and Code Availability

All raw and processed data from this study are openly available at https://osf.io/y2q3r/. The “WearableHRV” pipeline, used for statistical analysis, is open source and can be found here: https://github.com/Aminsinichi/wearable‐hrv/. This paper includes a verified computational reproducibility (see Quintero et al. 2024), confirmed through an independent CODECHECK process, which is an open science initiative to improve reproducibility (Nüst and Eglen 2021).

### 2.10 Supporting Information Overview

In the Table S1 summarizes the number of participants included in each condition and device analysis; Table S2 details signal quality categorized as acceptable, poor, or missing. Table S3 provides descriptive statistics (mean, standard deviation, maximum, and minimum) for RMSSD, HF, and mean HR across all conditions and devices. Table S4 presents MAAPE values with 95% confidence intervals for these metrics. Figures S4–S6 contain scatter plots for LnRMSSD, LnHF, and mean HR, including correlation coefficients, Bonferroni‐adjusted *p*‐values, and observation counts. Bland–Altman comparison plots for LnRMSSD, LnHF, and mean HR, along with biases, standard deviations, and LoAs, are shown in Figures S7–S12 and Table S5. Preprocessing methods are illustrated in Figures S1 and S2, with additional comparisons of raw ECG and PPG signals provided in Figure S13. Lastly, Figure S3 presents the RMS values of VU‐AMS acceleration data across all conditions. For conciseness of the current manuscript, all the results of the HF measures are included only in the Appendix S1.

## 3 Results

### 3.1 Signal Quality

Descriptive results of signal quality are presented in Table 1, where for each device and condition, the mean and standard deviation of the number of artifacts, the data quality (expressed as a percentage of poor and missing data), and the mean IBI detection rate (expressed as a percentage of detected IBIs divided by true IBIs) are reported. The mean and standard deviation of acceleration data based on Empatica's accelerometer are also incorporated into this table.

**TABLE 1:** Descriptive statistics of signal quality and acceleration: N artifacts: The detected number of artifacts, the mean, and the standard deviation. Data Quality: P stands for poor, and M stands for missing, expressed as a percentage for each condition.

	Condition/Device	sitting	arithmetic	recovery	standing	breathing	neurotask	walking	biking
N Artifacts (Mean & SD)	Kyto	0.0 (0.0)	0.03 (0.18)	0.0 (0.0)	0.0 (0.0)	0.0 (0.0)	0.0 (0.0)	0.0 (0.0)	0.0 (0.0)
	Rhythm	5.25 (16.43)	2.42 (3.95)	4.19 (10.63)	2.33 (5.99)	3.17 (6.05)	7.69 (19.36)	32.5 (21.92)	13.33 (12.81)
	HeartMath	0.85 (3.96)	3.08 (5.38)	0.33 (0.88)	0.25 (1.04)	0.82 (1.73)	0.85 (3.17)	1.3 (2.48)	16.12 (27.27)
	Empatica	7.15 (15.76)	42.44 (31.15)	11.18 (18.37)	16.29 (28.04)	9.65 (14.65)	25.62 (41.48)	82.03 (22.38)	84.9 (42.54)
	VU‐AMS	0.07 (0.35)	0.05 (0.22)	0.12 (0.56)	0.0 (0.0)	0.17 (0.38)	0.1 (0.37)	0.0 (0.0)	0.12 (0.56)
Data Quality	Kyto	*p* = 5% *M* = 25%	*p* = 7.5% *M* = 22.5%	*p* = 5% *M* = 22.5%	*p* = 12.5% *M* = 27.5%	*p* = 15% *M* = 20%	*p* = 7.5% *M* = 27.5%	*p* = 45% *M* = 20%	*p* = 35% *M* = 27.5%
	Rhythm	*p* = 15% *M* = 10%	*p* = 55% *M* = 10%	*p* = 20% *M* = 10%	*p* = 25% *M* = 10%	*p* = 27.5% *M* = 10%	*p* = 10% *M* = 10%	*p* = 77.5% *M* = 10%	*p* = 62.5% *M* = 10%
	HeartMath	*p* = 0% *M* = 0%	*p* = 0% *M* = 0%	*p* = 0% *M* = 0%	*p* = 0% *M* = 0%	*p* = 0% *M* = 0%	*p* = 0% *M* = 0%	*p* = 0% *M* = 0%	*p* = 40% *M* = 0%
	Empatica	*p* = 0% *M* = 15%	*p* = 27.5% *M* = 15%	*p* = 2.5% *M* = 15%	*p* = 10% *M* = 15%	*p* = 0% *M* = 15%	*p* = 5% *M* = 15%	*p* = 65% *M* = 20%	*p* = 60% *M* = 22.5%
Mean IBI Detection Rate	Kyto	97.24%	89.3%	92.7%	93.43%	92.04%	90.47%	64.92%	63.95%
	Rhythm	86.27%	61.3%	84.99%	77.16%	73.94%	84.5%	56.6%	58.39%
	HeartMath	99.74%	97.19%	99.56%	99.49%	99.04%	99.68%	98.41%	74.17%
	Empatica	99.22%	89.16%	98.52%	97.96%	99.75%	96.92%	88.98%	66.59%
Acceleration (Mean & SD)	Empatica	0.01 (0.0)	0.02 (0.01)	0.01 (0.0)	0.01 (0.0)	0.01 (0.0)	0.01 (0.0)	0.16 (0.03)	0.08 (0.02)

*Note:* The mean IBI detection rate is calculated by dividing the number of detected IBIs in a given device and condition by the number of true IBIs (based on the detected IBIs of the criterion device) in the same condition, multiplied by 100. Acceleration is in g units and is calculated by taking the euclidean distance of *x*, *y*, and *z* accelerometer channels from Empatica. Note that the duration of conditions is not equal: Sitting is 5 min, neurotask approximately 10 min, and the rest of the conditions each 3 min.

The detection rate and number of artifacts vary notably depending on the movement involved in a condition, with more movement‐intense conditions leading to a lower detection rate and more artifacts across devices. For example, in the recovery condition, Empatica exhibited artifacts with a mean and standard deviation of 11.18 (18.37), and a mean IBI detection rate of 99.22%. Whereas in the biking condition, it increased to 84.9 (42.54) artifacts, with a mean IBI detection rate of only 66.59%. A condition such as arithmetic, where participants are sitting still, yet have to listen and respond, which involves some subtle movement or muscle contraction, also had an effect on performance. For instance, Empatica had 42.44 (31.15) artifacts during arithmetic condition, with a mean IBI detection rate of 89.16%. Other devices also showed similar patterns: Kyto from 97.24% in sitting to 89.3% in arithmetic, and Rhythm from 86.27% in sitting to 61.3% in arithmetic. HeartMath, however, maintained a high IBI detection rate, from 99.74% in sitting to 97.19% in arithmetic condition.

**FIGURE 2:**
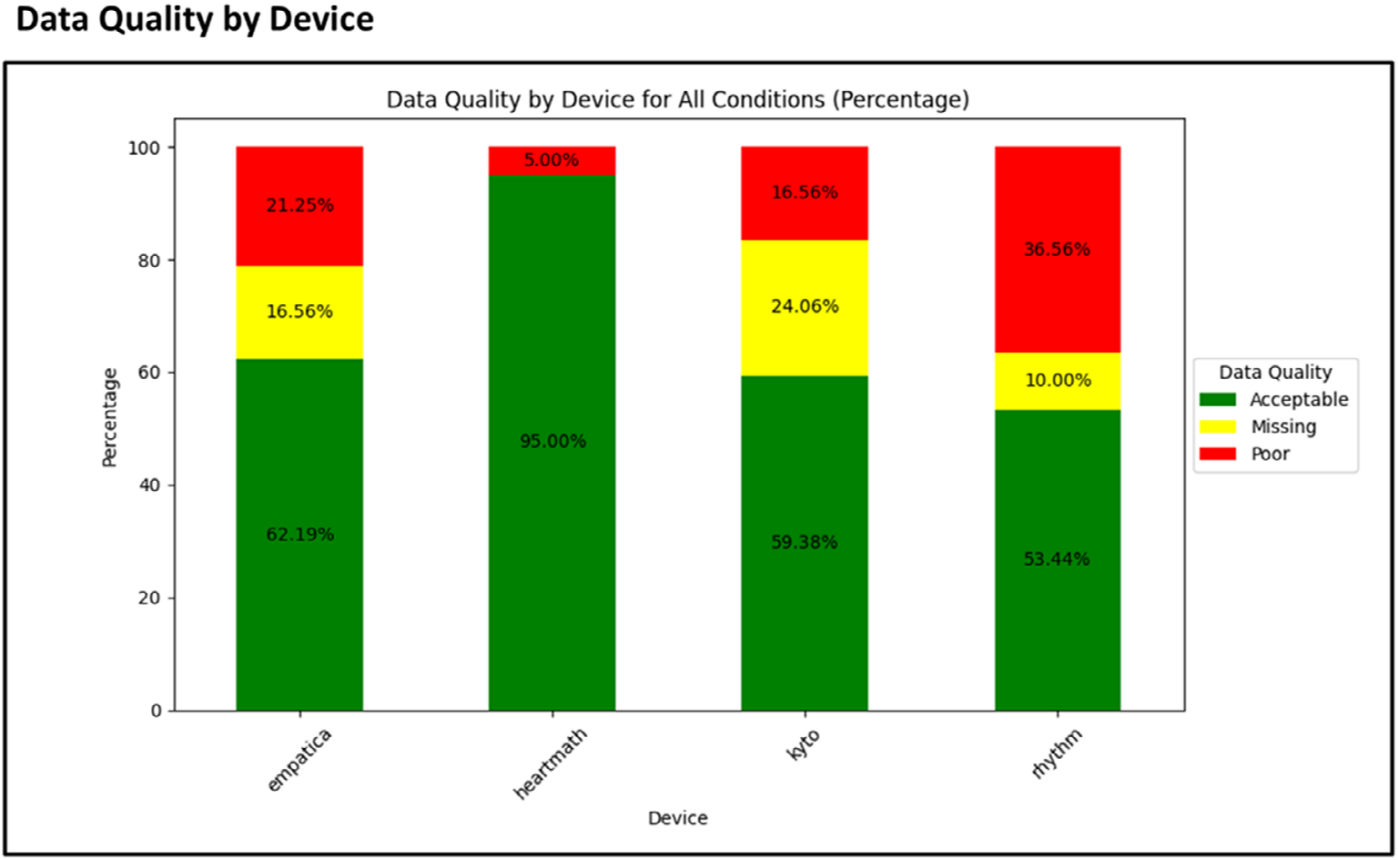
Data Quality by Device: The x‐axis represents each device. The y‐axis shows the percentage of signals classified as acceptable, missing, or of poor quality.

Figure 2 provides an overall overview of data quality for each device. The details of poor and missing data quality per device and condition are summarized in Table 1. These results should be interpreted in conjunction with one another. For example, Table 1 indicates that Kyto has 0 artifacts in the sitting condition, but this finding should be considered in conjunction with its missing rate, which is 25%. A similar approach should be taken when evaluating other devices. It is also important to consider the use case of each device when considering these results. For instance, the HeartMath shows completely acceptable signal quality in all the expected conditions for its use case (i.e., laboratory‐like conditions). Whereas taking Empatica as an example, in the ambulatory‐like conditions, only 15% of the signal is acceptable in walking, and 17.5% of the signal is acceptable in the biking condition.

**FIGURE 3:**
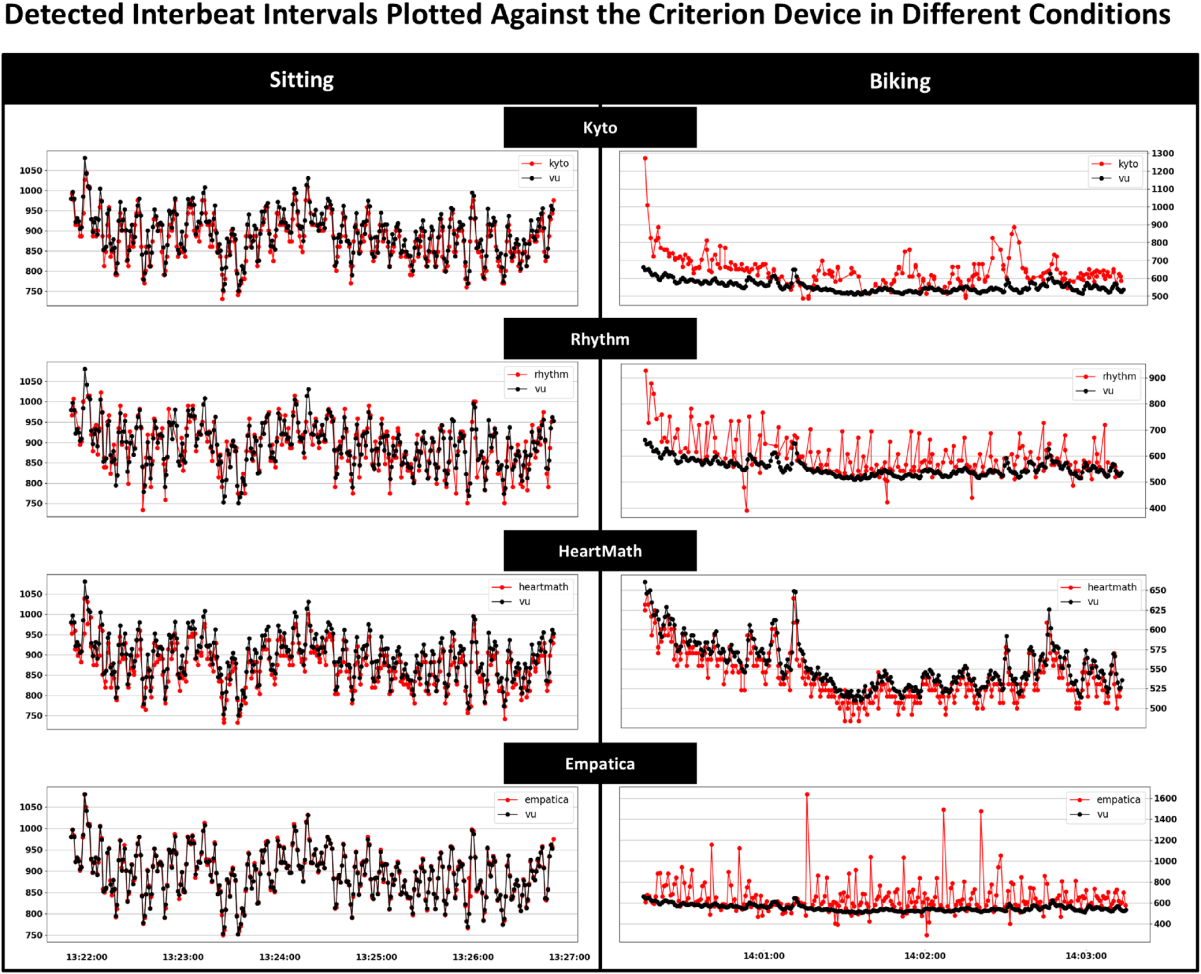
Detected Interbeat Intervals Plotted Against the Criterion Device: The graph shows two conditions: Sitting, and biking, from a single participant (P30). The black line in each subplot represents the IBIs measured by the criterion device (VU‐AMS); the red line represents the IBIs measured by the tested devices. The y‐axis of each subplot represents the IBIs in milliseconds, and the *x‐*axis for each subplot corresponds to time.

Figure 3 illustrates the detected IBI timeseries for each wearable for a single representative participant, plotted together with the time series as detected by the criterion device in two conditions, one representing a static laboratory‐based condition (sitting) and the other that represents an ambulatory‐like condition (biking).

### 3.2 Descriptive Statistics

**FIGURE 4:**
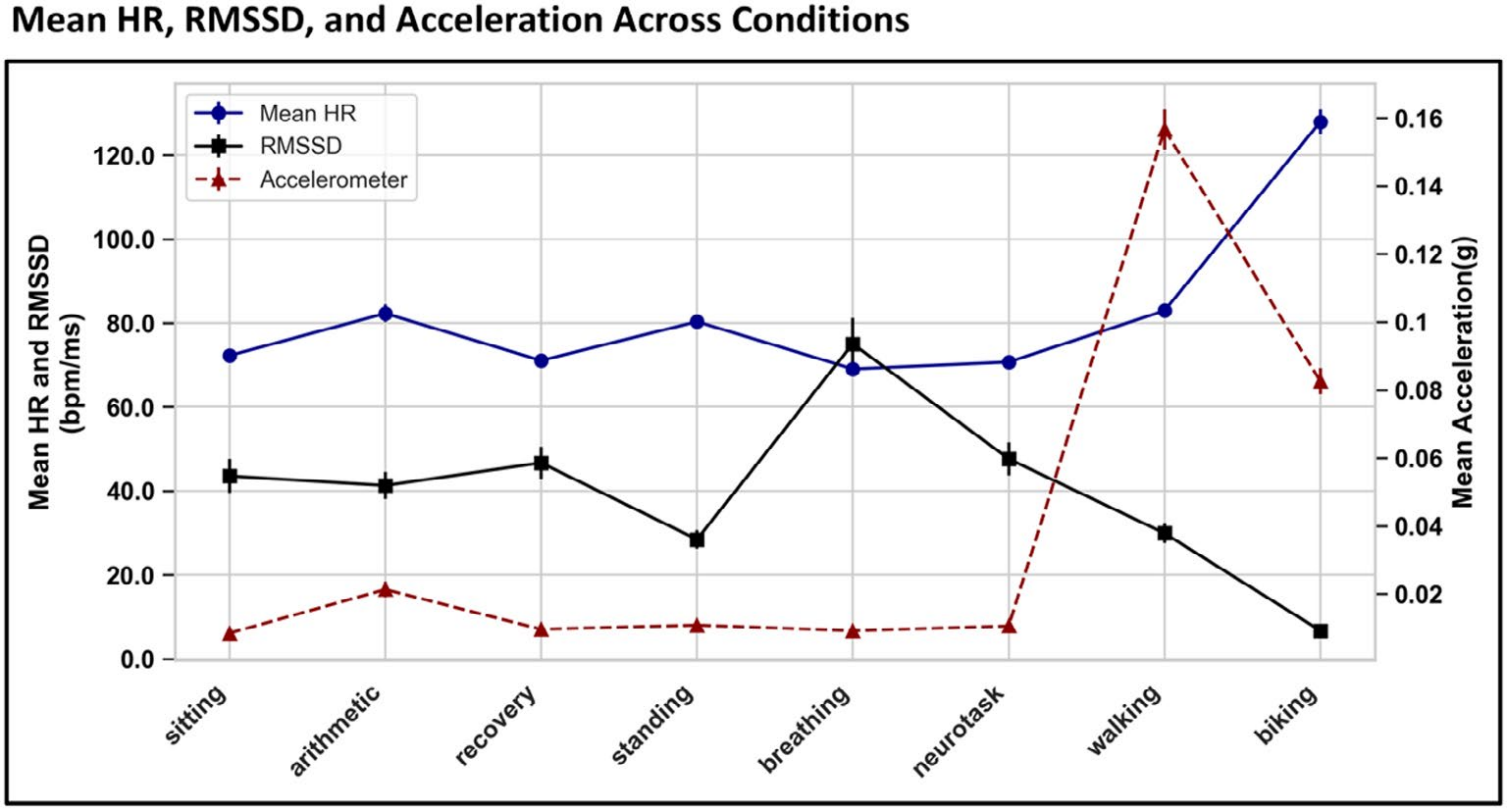
Mean HR, RMSSD, and Acceleration Across Conditions: Line graph illustrating the averaged mean HR (blue circles) and RMSSD (black squares) values across conditions on the left *y*‐axis, with accelerometer data from Empatica (red triangles) plotted on the right *y*‐axis. The *x*‐axis shows experimental conditions. Error bars show the standard error of the mean (SEM). The *y*‐axis for HR and RMSSD is in bpm/ms. The *y*‐axis for acceleration (in g) is derived from the Euclidean norm of *x*, *y*, and *z* components of the accelerometer data.

Figure 4 summarizes the mean HR, RMSSD (derived from VU‐AMS), and acceleration (derived from Empatica) across all experimental conditions, to provide an overview of heart rate variability and movement intensity for each condition. The error bars in these plots represent the standard error of the mean (SEM). The biking condition resulted in the highest mean HR (*M* = 127.93, SEM = 2.90), and the breathing condition in the lowest (*M* = 69.04, SEM = 1.71). Conversely, RMSSD values were highest during the breathing condition (*M* = 75.04, SEM = 6.06) and lowest during biking (*M* = 6.70, SEM = 0.81). The right y‐axis of this plot (corresponding to red triangle line) shows the movement associated with each condition, based on the Empatica's accelerometer data. The majority of movements were observed in the walking and biking conditions. Notably, arithmetic also involves some movement compared to other laboratory‐like conditions. The mean and the standard deviation of these values are summarized in Table 1. Note that given Empatica is worn on the non‐dominant wrist, it reflects the wrist acceleration rather than gross body movement. Figure S3 provides the acceleration data from VU‐AMS, which was worn on the left hip and may provide additional insight into body movement.

### 3.3 Device Performance Under Lab and Ambulatory‐Like Conditions

**FIGURE 5:**
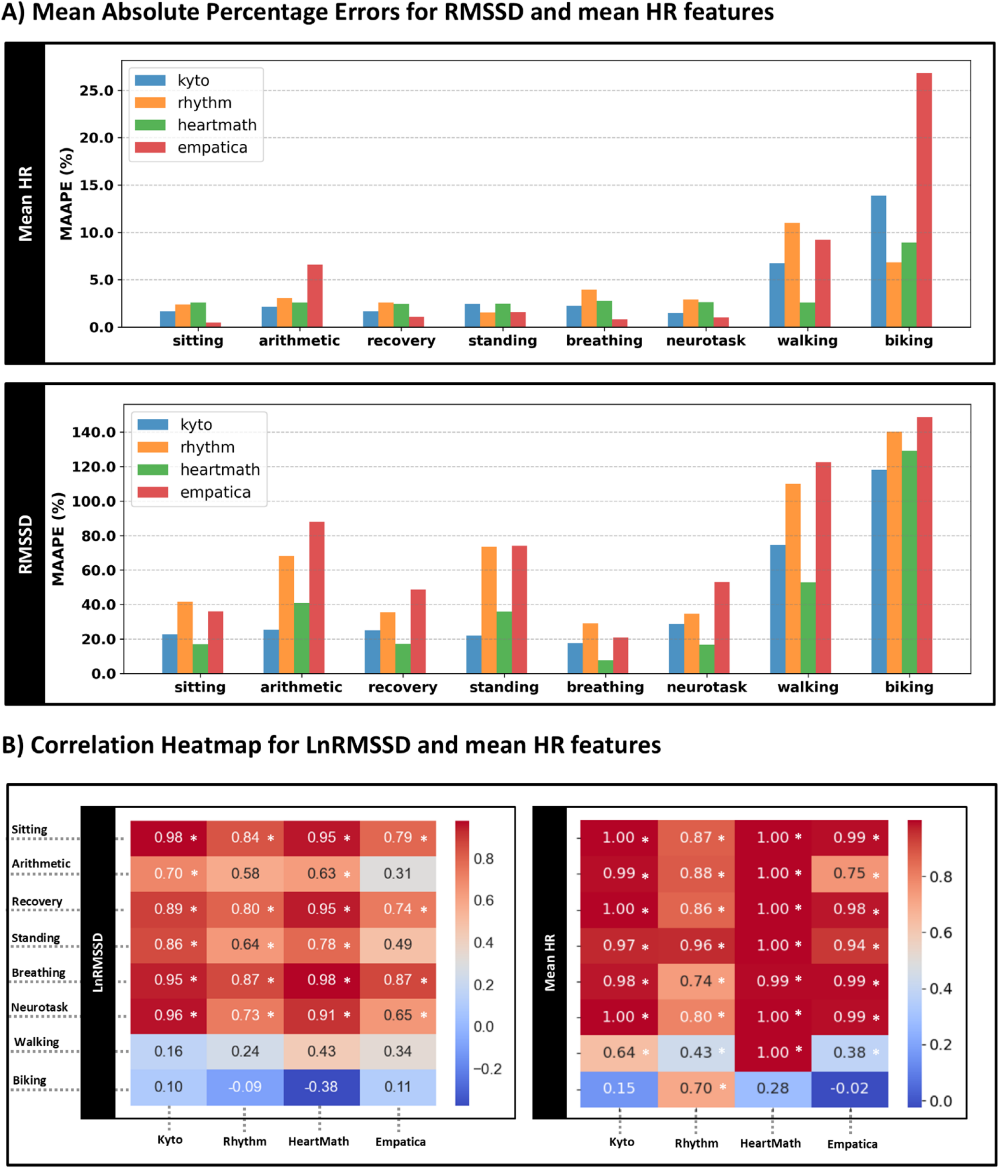
(A) Mean Arctangent Absolute Percentage Errors (MAAPE): The upper subplot displays values for mean HR; the lower subplot for RMSSD. The *y*‐axis indicates MAAPE values in percent. The *x*‐axis in each subplot represents the conditions. Each device is plotted with a color‐specific bar plot. (B) Correlation Heatmap for LnRMSSD and mean HR features: Each row in the heatmap represents a different condition. The values, separated for LnRMSSD (log‐transformed RMSSD) and mean HR, are displayed for all devices along the *x*‐axis. An asterisk (*) indicates statistical significance (Bonferroni‐corrected).

Figure 5A displays the MAAPE values for RMSSD and mean HR features. Heatmap plots illustrating the correlation coefficient values for regression analysis for each device and condition, compared with the corresponding condition in the criterion device, are shown in Figure 5B for LnRMSSD (log‐transformed RMSSD) and mean HR.

A summary of the results for the MAAPE, regression analysis, and Bland–Altman analyses for each device is presented in Tables 2, 3, 4, 5 below (details can be found in the Appendix S1). Note that Kyto, HeartMath, and Rhythm (when set to HRV mode) are intended for laboratory‐like settings, while Empatica is designed for both laboratory and ambulatory conditions. Therefore, even though the tables include results for all conditions, it should be noted that the poor performance of the consumer‐grade devices in ambulatory‐like conditions is expected, as it does not follow the intended use case.

**TABLE 2:** Analysis Results for Kyto: The results for MAAPE (Mean Arctangent Absolute Percentage Error), Pearson correlation (*r*), and biases (Bland–Altman analysis) are presented for mean heart rate and RMSSD.

	Measure	Sitting	Arithmetic	Recovery	Standing	Breathing	Neurotask	Walking	Biking
Mean HR	MAAPE (low CI, high CI)	1.67 (1.53, 1.8)	2.14 (1.36, 2.92)	1.68 (1.51, 1.85)	2.44 (0.96, 3.92)	2.25 (1.63, 2.87)	1.46 (1.26, 1.67)	6.72 (3.1, 10.34)	13.89 (8.24, 19.53)
Mean HR	*r* (low CI, high CI)	1.0 (0.999, 1.0)	0.99 (0.978, 0.995)	0.998 (0.997, 0.999)	0.97 (0.932, 0.984)	0.98 (0.965, 0.992)	0.999 (0.998, 0.999)	0.64 (0.37, 0.81)	0.15 (−0.23, 0.49)
Mean HR	Bias (low LoA, High LoA)	1.17 (1.05, 1.3)	1.43 (0.81, 2.06)	1.12 (0.94, 1.3)	1.65 (0.67, 2.62)	0.93 (0.37, 1.49)	1.02 (0.85, 1.19)	−3.21 (−6.92, 0.5)	−17.15 (−26.62, −7.68)
RMSSD	MAAPE (low CI, high CI)	22.78 (19.56, 26.01)	25.39 (14.69, 36.08)	25.05 (19.53, 30.56)	21.96 (13.73, 30.19)	17.51 (13.76, 21.26)	28.66 (25.24, 32.08)	74.41 (55.46, 93.36)	118.12 (102.82, 133.43)
LnRMSSD	*r* (low CI, high CI)	0.98 (0.95, 0.99)	0.7 (0.46, 0.84)	0.89 (0.78, 0.94)	0.86 (0.72, 0.93)	0.95 (0.89, 0.97)	0.96 (0.91, 0.98)	0.16 (−0.2, 0.48)	0.1 (−0.28, 0.45)
LnRMSSD	Bias (low LoA, High LoA)	−0.27 (−0.32, −0.22)	0.01 (−0.14, 0.16)	−0.25 (−0.34, −0.16)	−0.02 (−0.14, 0.09)	−0.21 (−0.27, −0.15)	−0.36 (−0.42, −0.31)	0.79 (0.49, 1.09)	1.73 (1.33, 2.13)

*Note:* For correlation and MAAPE, parentheses show the low and high 95% confidence intervals. For biases, parentheses indicate the low and high levels of agreement (LoA). LnRMSSD stands for log‐transformed RMSSD.

**TABLE 3:** Analysis Results for Rhythm: The results for MAAPE (Mean Arctangent Absolute Percentage Error), Pearson correlation (*r*), and biases (Bland–Altman analysis) are presented for mean heart rate and RMSSD.

	Measure	Sitting	Arithmetic	Recovery	Standing	Breathing	Neurotask	Walking	Biking
Mean HR	MAAPE (low CI, high CI)	2.39 (−0.22, 5.00)	3.07 (1.03, 5.11)	2.59 (−0.10, 5.28)	1.52 (0.19, 2.85)	3.94 (0.40, 7.49)	2.92 (−0.64, 6.47)	11.01 (6.61, 15.40)	6.85 (3.44, 10.26)
Mean HR	*r* (low CI, high CI)	0.87 (0.77, 0.93)	0.88 (0.78, 0.94)	0.86 (0.74, 0.93)	0.96 (0.93, 0.98)	0.74 (0.54, 0.86)	0.80 (0.64, 0.89)	0.43 (0.12, 0.67)	0.70 (0.49, 0.84)
Mean HR	Bias (low LoA, High LoA)	1.24 (−0.43, 2.91)	−1.35 (−3.50, 0.81)	1.19 (−0.53, 2.91)	0.65 (−0.36, 1.66)	1.26 (−1.04, 3.57)	1.47 (−0.69, 3.64)	5.06 (0.26, 9.86)	−8.36 (−13.65, −3.07)
RMSSD	MAAPE (low CI, high CI)	41.62 (31.39, 51.85)	68.09 (55.27, 80.91)	35.50 (24.54, 46.45)	73.62 (61.60, 85.63)	29.08 (20.49, 37.67)	34.56 (23.95, 45.17)	110.16 (98.97, 121.35)	140.21 (133.92, 146.49)
LnRMSSD	*r* (low CI, high CI)	0.84 (0.70, 0.91)	0.58 (0.31, 0.76)	0.80 (0.64, 0.89)	0.64 (0.39, 0.80)	0.87 (0.75, 0.93)	0.73 (0.53, 0.85)	0.24 (−0.09, 0.53)	−0.09 (−0.41, 0.24)
LnRMSSD	Bias (low LoA, High LoA)	0.38 (0.28, 0.49)	0.66 (0.51, 0.81)	0.32 (0.21, 0.43)	0.72 (0.57, 0.86)	0.23 (0.14, 0.32)	0.28 (0.17, 0.40)	1.25 (1.08, 1.43)	2.34 (2.07, 2.61)

*Note:* For correlation and MAAPE, parentheses show the low and high 95% confidence intervals. For biases, parentheses indicate the low and high levels of agreement (LoA). LnRMSSD stands for log‐transformed RMSSD.

**TABLE 4:** Analysis Results for HeartMath: The results for MAAPE (Mean Arctangent Absolute Percentage Error), Pearson correlation (*r*), and biases (Bland–Altman analysis) are presented for mean heart rate and RMSSD.

	Measure	Sitting	Arithmetic	Recovery	Standing	Breathing	Neurotask	Walking	Biking
Mean HR	MAAPE (low CI, high CI)	2.60 (2.34, 2.86)	2.58 (2.18, 2.99)	2.44 (2.31, 2.56)	2.46 (2.42, 2.5)	2.75 (2.34, 3.16)	2.63 (2.41, 2.85)	2.59 (2.46, 2.73)	8.91 (5.35, 12.46)
Mean HR	*r* (low CI, high CI)	0.999 (0.998, 0.999)	0.998 (0.996, 0.999)	0.999 (0.999, 1.0)	1.0 (0.999, 1.0)	0.995 (0.99, 0.997)	0.999 (0.998, 0.999)	0.999 (0.999, 0.999)	0.28 (−0.04, 0.54)
Mean HR	Bias (low LoA, High LoA)	1.85 (1.71, 2.0)	2.06 (1.81, 2.32)	1.73 (1.61, 1.86)	1.97 (1.89, 2.06)	1.91 (1.59, 2.22)	1.85 (1.7, 2.0)	2.15 (2.0, 2.31)	−10.03 (−16.38, −3.68)
RMSSD	MAAPE (low CI, high CI)	17.03 (11.33, 22.73)	40.93 (28.9, 52.96)	17.07 (12.3, 21.84)	35.94 (24.99, 46.88)	7.7 (5.21, 10.19)	16.65 (11.2, 22.1)	52.75 (39.59, 65.92)	129.09 (117.62, 140.56)
LnRMSSD	*r* (low CI, high CI)	0.95 (0.91, 0.98)	0.63 (0.40, 0.79)	0.95 (0.90, 0.97)	0.78 (0.63, 0.88)	0.98 (0.96, 0.99)	0.91 (0.83, 0.95)	0.43 (0.14, 0.66)	−0.38 (−0.62, −0.07)
LnRMSSD	Bias (low LoA, High LoA)	0.12 (0.06, 0.18)	0.37 (0.25, 0.5)	0.11 (0.05, 0.16)	0.32 (0.21, 0.42)	−0.01 (−0.05, 0.02)	0.08 (0.01, 0.14)	0.5 (0.36, 0.64)	2.14 (1.81, 2.47)

*Note:* For correlation and MAAPE, parentheses show the low and high 95% confidence intervals. For biases, parentheses indicate the low and high levels of agreement (LoA). LnRMSSD stands for log‐transformed RMSSD.

**TABLE 5:** Analysis Results for Empatica: The results for MAAPE (Mean Arctangent Absolute Percentage Error), Pearson correlation (*r*), and biases (Bland–Altman analysis) are presented for mean heart rate and RMSSD.

	Measure	Sitting	Arithmetic	Recovery	Standing	Breathing	Neurotask	Walking	Biking
Mean HR	MAAPE (low CI, high CI)	0.49 (−0.09, 1.06)	6.6 (3.74, 9.46)	1.09 (0.36, 1.81)	1.58 (0.28, 2.88)	0.8 (0.28, 1.33)	1.01 (0.35, 1.67)	9.22 (6.37, 12.07)	26.83 (21.96, 31.69)
Mean HR	*r* (low CI, high CI)	0.99 (0.98, 0.99)	0.75 (0.56, 0.87)	0.98 (0.97, 0.99)	0.94 (0.89, 0.97)	0.99 (0.99, 1.00)	0.99 (0.98, 0.99)	0.38 (0.04, 0.65)	−0.02 (−0.38, 0.33)
Mean HR	Bias (low LoA, High LoA)	−0.15 (−3.01, 2.71)	−5.79 (−23.33, 11.76)	−0.35 (−4.05, 3.36)	−0.63 (−7.59, 6.34)	0.06 (−2.07, 2.20)	−0.44 (−3.58, 2.70)	−5.38 (−24.78, 14.02)	−38.00 (−80.65, 4.65)
RMSSD	MAAPE (low CI, high CI)	36.16 (23.7, 48.62)	87.98 (73.79, 102.17)	48.62 (35.4, 61.85)	74.01 (58.83, 89.19)	20.98 (11.72, 30.23)	52.92 (38.41, 67.44)	122.58 (115.73, 129.44)	148.68 (146.61, 150.76)
LnRMSSD	*r* (low CI, high CI)	0.79 (0.62, 0.89)	0.31 (−0.03, 0.59)	0.74 (0.53, 0.86)	0.49 (0.18, 0.71)	0.87 (0.75, 0.93)	0.65 (0.40, 0.81)	0.34 (−0.02, 0.61)	0.11 (−0.25, 0.45)
LnRMSSD	Bias (low LoA, High LoA)	0.33 (0.20, 0.46)	0.92 (0.74, 1.11)	0.45 (0.32, 0.58)	0.76 (0.57, 0.94)	0.18 (0.09, 0.27)	0.49 (0.34, 0.64)	1.45 (1.29, 1.61)	2.79 (2.55, 3.04)

*Note:* For correlation and MAAPE, parentheses show the low and high 95% confidence intervals. For biases, parentheses indicate the low and high levels of agreement (LoA). LnRMSSD stands for log‐transformed RMSSD.

#### 3.3.1 Kyto

As noted earlier (Figure 2), the rate of missing data in Kyto was high, which for most use cases will be unacceptable. In the limited instances where data was available, it correlated almost perfectly with the criterion in terms of mean HR in all laboratory‐like conditions, with relatively small biases and MAAPE.

The correlation for RMSSD in sitting, breathing, and neurotask conditions was also very high (*r* values above 0.95). It became slightly worse in the standing condition (*r* = 0.86) and was lowest in the arithmetic task (*r* = 0.7).

#### 3.3.2 Rhythm

Rhythm on HRV‐mode is tailored for measurement in resting‐state conditions. Yet, the data quality was compromised in these conditions, such that in sitting, only 75% of the data had acceptable quality, which was also reflected in its performance in terms of agreement between mean HR and RMSSD with the criterion. This device showed the highest correlation (*r* = 0.95), the lowest MAAPE (1.52), and bias (0.65) in the standing condition for mean HR. Despite this exception, the correlations for other conditions, such as sitting, arithmetic, recovery, and neurotask, all fell within the range of 0.80. In the breathing condition, the correlation dropped to 0.74, with a MAAPE of 3.94 and a bias of 1.26 beats.

For RMSSD, its performance varied, with the highest correlation observed in the breathing condition (*r* = 0.87), followed by sitting (*r* = 0.84). In the neurotask (*r* = 0.73), standing (*r* = 0.64), and arithmetic (*r* = 0.58) conditions, the correlation worsened.

#### 3.3.3 HeartMath

HeartMath's interbeat interval time series had 100% acceptable quality in laboratory‐like conditions intended for its use case. In the majority of conditions, HeartMath correlated almost perfectly with the criterion device in terms of mean HR, with small MAAPE and biases. This is even evident in the walking condition, which this device is presumably not designed to be used in, but still with a correlation of 0.99, MAAPE of 2.59, and a bias of 2.15, it shows a very accurate agreement.

For RMSSD, the correlation is the highest in sitting, recovery, breathing, and neurotask conditions (*r* above 0.90), although the MAAPE and biases vary in these conditions. In the arithmetic task, the correlation falls to 0.63, and the MAAPE becomes large (40.93).

#### 3.3.4 Empatica

Empatica was the only research‐grade device in this study that was intended for ambulatory‐like recordings, yet its signal quality specifically in these conditions was compromised, either due to the presence of artifacts, missing data, or missed beats. For mean HR, Empatica could accurately measure it during sitting, recovery, breathing, neurotask, and standing conditions, evident by a very high correlation coefficient (*r* above 0.90), and low MAAPE and biases. However, in the arithmetic condition, the correlation worsens (0.75), and the MAAPE (6.6) and bias (−5.79) become larger. This gets noticeably worse in ambulatory conditions, such that the correlation between Empatica and the criterion in the biking condition becomes zero (−0.02), with a − 38 beats bias in estimating the heart rate, and a MAAPE of 26.83.

For RMSSD, Empatica does not show as high an agreement compared with the criterion in any of the conditions. The highest correlation between Empatica and the criterion is in the breathing condition (*r* = 0.87). In all other laboratory conditions, the correlation decreases, the MAAPE increases, and the biases become larger. In the arithmetic condition, the correlation is low (0.31), and the MAAPE is notably large (87.98). Even in the standing condition, *r* = 0.49 and MAAPE = 74.01. The accuracy diminishes as the movement intensity progresses, such that it does not show acceptable agreement in ambulatory conditions, worsening in the biking condition with a 0.11 correlation and a very large MAAPE (148.68).

## 4 Discussion

In this study, we evaluated the accuracy of four heart rate PPG wearables for HR and HRV indices. Three of these devices were consumer‐grade (Kyto, Rhythm, HeartMath), and one was the research‐grade Empatica EmbracePlus. We conducted simultaneous recordings with these devices and compared their data to an ECG device, the VU‐AMS. Our experimental conditions spanned both laboratory and ambulatory‐like settings, aiding in identifying the circumstances under which these devices demonstrate optimal performance. The consumer‐grade devices in this study are primarily marketed for use in laboratory‐like conditions, such as delivering biofeedback and measuring resting‐state HRV, whereas Empatica is designed to perform both in laboratory and ambulatory conditions. Our objective in validating these wearables was not only to provide detailed performance metrics for each device under various conditions but also to draw clear conclusions for psychophysiological studies that aim to measure HR and HRV using PPG‐based wearables.

### 4.1 Main Findings

Among the devices tested in our study, Kyto frequently failed to connect and transfer IBIs, encountering issues in about a quarter of the instances. Rhythm generally displayed larger errors and biases, along with lower correlations. Empatica's accuracy declined in any condition deviating from a resting state. HeartMath demonstrated the best performance, exhibiting the highest signal quality, strongest correlation with the criterion, and the lowest errors, biases and levels of agreement.

Sitting, recovery, breathing, and neurotask shared mutual characteristics, as in all of these laboratory‐like conditions participants remained seated with no movement or speech. This similarity led to broadly consistent device agreement when compared with the criterion across these conditions. All devices correlated strongly with the criterion for mean HR, exhibiting small errors and biases. However, Rhythm showed higher biases and lower correlations in these conditions compared to other devices. For RMSSD, HeartMath displayed the best performance with the highest correlation, highest signal quality, lowest error, and least bias. Contrary to our initial expectations, Empatica did not outperform consumer‐grade devices under these conditions.

The remaining two laboratory‐like conditions, arithmetic and standing, provide further insights into the performance of the PPG devices. What makes these conditions unique is that they involve little to no movement (Figure 4), yet the slight deviation from a resting state seems to still affect the PPG signal, evident with higher errors and lower agreement. For mean HR, devices generally maintain agreement as in other laboratory conditions, except for Empatica, which shows decreased accuracy in the arithmetic task with a correlation of *r* = 0.75 and an underestimation bias of −5.75 beats, with large levels of agreement (−23.33, 11.76). For RMSSD, all devices tend to perform worse. For instance, Empatica shows correlations of *r* = 0.31 in arithmetic and *r* = 0.49 in standing. HeartMath, on the other hand, shows slightly better performance in these conditions with correlations of 0.63 and 0.78, respectively, alongside smaller biases and MAAPE values. These two conditions highlight an important limitation of PPG‐based HRV metrics, where even slightly deviating from a resting state compromises the data quality.

Among the tested devices, Empatica is the only one expected to perform accurately in ambulatory conditions. Despite our expectations, it performed poorly in slow‐paced walking and stationary biking conditions, showing low accuracy for both RMSSD and mean HR. It showed negligible correlation, large errors and biases, and wide limits of agreement. Furthermore, signal quality was poor, with only 15% considered acceptable during walking and 17.5% during biking (Table 1). Contrarily, HeartMath, although not intended for such conditions, showed a perfect correlation with the criterion in the walking condition for mean HR. It displayed a small MAAPE of 2.59% and minor biases of 2.15, maintaining 100% acceptable signal quality.

At the outset, we formulated four hypotheses. The first hypothesis proposed that PPG‐based wearables would yield higher agreement for HR compared with HRV metrics (specifically RMSSD and HF‐HRV). We found that all devices performed better at detecting HR compared with HRV (Hu et al. 2024; Ishaque, Khan, and Krishnan 2021; Sarhaddi et al. 2022). The calculation of RMSSD and HF‐HRV values is particularly sensitive, as it relies on the precise detection of each IBI. Missing a few IBIs or having data dominated by artifacts can significantly affect these values. Different preprocessing techniques, such as the Karlsson method we used, can to some degree help remove artifacts, yet it has its own limitations, specifically when adjacent artifacts are present. On the other hand, the way mean HR is calculated for each condition (by dividing the mean IBIs by 60,000) allows for a more accurate capture of HR. This was particularly evident in more static conditions. In line with our second hypothesis, we anticipated higher agreement with the criterion in laboratory‐like conditions compared to ambulatory‐like conditions. This was true for both HR and HRV metrics. Specifically, in static conditions such as sitting, all devices exhibited a small MAAPE relative to the criterion but, this accuracy diminished as movement increased. An example of this can be seen in the MAAPE for mean HR in Empatica, which ranges from 0.49% in sitting to 26.83% in biking, whereas for RMSSD, it goes from 36.16% in sitting (larger errors even at baseline) to 148.68% in biking.

Our third and fourth hypotheses proposed that the Empatica device, marketed as a research‐grade wearable, would outperform the other devices in terms of HR and HRV metrics in both laboratory‐like and ambulatory‐like conditions. Contrary to our expectations, the results indicate the opposite pattern. Our findings show that the research‐grade Empatica device was not superior to consumer‐grade devices in laboratory‐like conditions and, in some instances, was even inferior. Its performance in ambulatory‐like conditions also revealed low agreement with the criterion ECG. This observation is consistent with findings from other studies, such as those by Bent et al. (2020) and aligns with prior assessments of its predecessor the Empatica E4, especially in conditions involving movement (Hu et al. 2024; Menghini et al. 2019; Van Voorhees et al. 2022). It is crucial for psychophysiological researchers to be aware of potential validity concerns, such as those presented in our study, given that research‐grade PPG‐based wearables, like Empatica, are widely marketed and employed in ambulatory research for continuously monitoring autonomic nervous system correlates such as HRV.

### 4.2 Future Recommendations

Our results, in conjunction with similar findings, offer clear recommendations for future studies aiming to use PPG wearables in the context of psychophysiological research:

1. If researchers are particularly interested in identifying HRV metrics such as RMSSD and HF‐HRV, which are among the most accessible non‐invasive proxies of the parasympathetic arm of the autonomic nervous system, it is recommended to use an ECG device to ensure a high signal‐to‐noise ratio, as proposed by numerous other studies (e.g., Hinde, White, and Armstrong 2021; Hu et al. 2024; Laborde, Mosley, and Thayer 2017; Quigley et al. 2024; Speer et al. 2020). This recommendation becomes even more critical when the research design includes conditions that deviate from a resting state. Such conditions may include ambulatory‐like settings, as well as experimental manipulations like posture changes or cognitive tasks, as demonstrated in our study. Even activities such as typing on a keyboard have been shown to affect the performance of these devices (Hu et al. 2024; Menghini et al. 2019). For mean heart rate (or its inverse, mean heart period), our results indicate that biases are considerably lower and agreement is much higher, particularly under static conditions.
2. Related to point one, it is important to note that devices are not the same. They vary in terms of hardware (e.g., the types of LEDs), the location where they can be worn (sensor placement), the sampling rate of the PPG signal, and software (e.g., proprietary peak detection algorithms). All these factors contribute to the sources of artifacts discussed above, and researchers are encouraged to consider them carefully when selecting a wearable device for their studies. This decision depends heavily on several considerations, including the experimental design (e.g., static laboratory‐based conditions versus dynamic ambulatory conditions), the specific signal and level of precision required (e.g., mean HR over a condition versus RMSSD or HF‐HRV), the research question being addressed, and the tradeoff between validity, reliability, and participant comfort. Given that the devices used in our study vary in more than one of these factors, it is difficult to draw specific conclusions about any one factor in isolation. However, some general conclusions can still be drawn when considering our findings alongside those of other studies. Theses factors should be carefully considered when selecting a device for a specific sample, and a specific use‐case:
  First, the location of the device impacts data quality (Armañac‐Julián et al. 2022; Maeda, Sekine, and Tamura 2011; Rajala, Lindholm, and Taipalus 2018), as it determines skin contact, the susceptibility to misplacement due to movement, and the muscle‐induced artifacts specific to the site. In our study, sensors on the earlobe appeared to outperform those on the wrist and arm. Second, PPG fiducial point detection is inherently more challenging compared to the sharp R peaks of ECG signals. This challenge is compounded by distortions from movement, and posture changes. In addition to this, the device's sampling rate is another critical factor (Laborde, Mosley, and Thayer 2017; Shaffer, McCraty, and Zerr 2014), especially for metrics such as RMSSD and HF‐HRV, which require precise systolic peak detection. For instance, comparing VU‐AMS (sampling rate: 1000 Hz) to Empatica (sampling rate: 64 Hz), the former captures data every millisecond while the latter samples every 15.626 ms. As heart periods shorten during higher intensity activity, the chance of missing or misplacing a peak increases with lower sampling rates. Third, the peak detection algorithms and signal pre‐processing methods used by devices vary, leading to different results. For example, as shown in the Supporting Information (Figure S13), applying a different preprocessing approach than the one used by the Empatica device itself produced different interbeat‐interval time series. Fourth, the type of LED used in the device has its own advantages and disadvantages, which researchers need to weigh based on their specific research objectives and target population. For instance, green light (492–577 nm) is more readily absorbed by blood hemoglobin and is less prone to motion artifacts. However, it has lower skin penetration and its performance is more affected by skin color, threatening validity for diverse populations. In contrast, red and infrared wavelengths are more robust in regard to variations in skin color but in turn are more susceptible to motion artifacts (Kim and Baek 2023; Lee et al. 2013; Spigulis et al. 2007).
3. In addition to these device‐specific factors mentioned above, participant‐related factors also influence data quality. There might be an individual difference between the performance of PPG‐based wearables and the HR and HRV metrics derived from them. One of these device‐participant interactions can be attributed to skin, as discussed above, as it is well‐documented that PPG sensors have limitations with darker skin tones and variations in skin thickness and composition (Fine et al. 2021; Hill et al. 2015). Moreover, the BMI of participants can also affect the quality of PPG signals (Yi et al. 2013), as the presence of adipose tissue influences the ability of the sensor to capture reliable data. Our study noted instances where the performance of these wearables differed among participants during data collection. Specifically, there were cases where a device failed to detect any signal at various body locations on one participant, yet functioned correctly when immediately tested on the researcher. This highlights the necessity of testing these devices on a diverse group of participants with varying BMIs, wrist circumferences, and skin colors, to conclusively assess the validity of a device.
4. Psychophysiological researchers are encouraged to select devices that have undergone external validation and demonstrated optimal performance for the metrics and conditions relevant to their study interests, with known strengths and limitations (Alugubelli, Abuissa, and Roka 2022; Charlton et al. 2023; Cosoli, Antognoli, and Scalise 2023; D'Angelo et al. 2023). Our results, along with similar findings we reviewed, demonstrate that the label “research‐grade” does not guarantee superior data quality. Currently, no regulations or standardized protocols define the criteria for labeling a device as research‐grade, leaving no guarantee that such devices provide reliable data quality. As a result, claims made by manufacturers should be approached with caution, given many marketing claims made by device manufacturers lack scientific evaluation, and external validation studies to support such claims are often absent. Promising initiatives, such as the Digital Medicine Society (Goldsack et al. 2020) and Stress in Action (https://stress‐in‐action.nl/), aim to build comprehensive databases of sensors and their use cases in clinical and research contexts. Additionally, researchers are advised to prioritize wearables that provide access to raw data, such as interbeat intervals for cardiac measurements, rather than relying solely on aggregated or precalculated metrics. This approach allows researchers to evaluate the device's accuracy against a gold‐standard ECG during a pilot phase before making large‐scale purchases or implementing the device in research studies. The validation pipeline used in this study has been detailed in an open‐source Python package (Sinichi, Gevonden, and Krabbendam 2024), hosted on GitHub (https://github.com/Aminsinichi/wearable‐hrv), which will hopefully facilitate future validation studies and encourages contributions toward developing a standardized framework for validating and reporting HR and HRV metrics (Nelson et al. 2020; Quigley et al. 2024).
5. At the very least, researchers are encouraged to provide a report on the signal quality of the wearable they are using. This can be done, for instance, by reporting the number of artifacts as a fraction of the detected beats (a signal‐to‐noise ratio). Such reporting helps in understanding the extent to which the data is usable and the circumstances that provided sufficient signal quality. To facilitate this, researchers are encouraged to use wearables that provide the raw data (raw PPG, or at least, detected IBIs) and not rely blindly on aggregated estimates calculated by the wearables.
6. Finally, our results highlight an important limitation of devices' peak detection algorithm that researchers need to take into account. We show that relying on the detected interbeat intervals from a device's internal peak detection algorithm, and then using (semi‐) automated methods of pre‐processing and cleaning the signal (e.g., the Karlsson method we used), does not guarantee high signal quality or strong agreement with a gold standard. Researchers are encouraged to incorporate visual inspection into their pre‐processing pipeline (Quigley et al. 2024; Shaffer and Ginsberg 2017) and reject data epochs with poor quality. Alternatively, if the nature of the research does not permit this, one can still gain valuable insights by measuring additional signals (e.g., accelerometer) and considering the exclusion of epochs with higher movement intensity, which usually leads to poor data quality and more artifacts.

### 4.3 Limitations

There are some limitations that need to be taken into account while interpreting our results. First, it is known from studies that one source of inaccuracy in PPG wearables might be skin color (Hill et al. 2015). In our study, we recruited participants with minor variation in skin color, the majority being light‐skinned, therefore it was not possible for us to test this aspect. In addition, the age range of our sample is limited to a narrow scope, centered on young adulthood. This is important to consider, as pediatric and senior populations, which are also of interest in psychophysiological research, have specific characteristics which may result in variation in device performance. For instance, in children, sensor placement can lead to variations in obtaining the blood pulse volume signal from PPG devices due to smaller wrist circumference, while aging can affect vascularization, potentially influencing the PPG waveform (Derraik et al. 2014; Fine et al. 2021). Second, the user manual for the Rhythm explicitly instructs that when the recording setting is set to HRV mode, measurements are only possible under static conditions. Kyto and HeartMath were also developed, not necessarily for ambulatory settings, but primarily for resting‐state measurements and biofeedback. Nonetheless, we tested all these devices in ambulatory conditions as well. However, in reporting the results, we cautioned the reader to mainly focus on the specific use of each wearable. Third, we did not exclude poor signal quality data for statistical analysis. Although doing so would increase the agreement between a device and the criterion, such agreement is rather artificial, given that in real‐life research, there is no criterion ECG present to compare the PPG‐derived metrics against and define and exclude poor segments of signal. Fourth, for Empatica, we only used systolic peaks automatically detected by the device. Empatica does offer the raw PPG signal, and using different algorithms to detect peaks might yield better results. However, since most researchers rely on the detected beats and previous studies with the E4 that scored the raw PPG did not yield a noticeable difference (Menghini et al. 2019), we decided not to pursue this. Finally, the pre‐processing approach used in our study may influence the results. Without access to raw signals, we employed thresholding to identify potential outlier IBIs. These detected outliers were then removed and the gaps were filled through linear interpolation. It is crucial to consider that different methods for handling outliers and cleaning data could lead to slightly varied outcomes, especially for HRV measures; less stringent pre‐processing or not interpolating values will inevitably lead to inflated RMSSD and HF‐HRV values. However, this becomes particularly relevant in the presence of numerous artifacts, which typically suggest poor signal quality and inherently low agreement by itself. Related to this point, it is important to mention that the two metrics used in the current study, namely RMSSD and HF‐HRV, are particularly sensitive to artifacts and variation in pre‐processing approaches. Not all HRV metrics are created equal, and other metrics, such as SDNN, may be less sensitive to phasic artifacts, especially over longer experimental epochs.

### 4.4 Conclusion

Our results have implications for psychophysiological researchers aiming to measure HR and HRV with PPG‐based wearables. We showed that noise‐driving factors, such as movement present in ambulatory‐like conditions or certain laboratory‐like conditions, impact PPG signal quality. In conditions such as performing a mental arithmetic task, standing up, slow‐paced walking, and stationary biking, all PPG‐based devices exhibited lower agreement with the criterion ECG, specifically for HRV (RMSSD, HF‐HRV). Several other studies have consistently shown that PPG devices perform poorly under conditions with movements (Bent et al. 2020; Castaneda et al. 2018; Null and Jafari 2015; Dobbs et al. 2019; Georgiou et al. 2018; Hu et al. 2024; Jo et al. 2016; Lu et al. 2009; Peake, Kerr, and Sullivan 2018; Schäfer and Vagedes 2013; Shcherbina et al. 2017; Zhang et al. 2019). Finally, our results suggest that for laboratory‐like conditions, HeartMath showed the highest agreement for HR and HRV with the criterion. Empatica, even though marketed as a research‐grade ambulatory device, did not show superior performance to consumer‐grade devices in this study.

## Author Contributions

**Mohammadamin Sinichi:** conceptualization, formal analysis, investigation, methodology, software, visualization, writing – original draft, writing – review and editing. **Martin J. Gevonden:** conceptualization, methodology, supervision, writing – review and editing. **Lydia Krabbendam:** conceptualization, funding acquisition, methodology, supervision, writing – review and editing.

## Conflicts of Interest

The authors declare no conflicts of interest.

## Supporting information


Appendix S1.

## Data Availability

All raw and processed data from this study are openly available at https://osf.io/y2q3r/. The “WearableHRV” pipeline, used for statistical analysis, is open source and can be found here: https://github.com/Aminsinichi/wearable‐hrv/. This paper includes a verified computational reproducibility (see Quintero et al. 2024), confirmed through an independent CODECHECK process, which is an open science initiative to improve reproducibility (Nüst and Eglen 2021).
