# Supplementary material for: Quality in Question: Assessing the Accuracy of Four Heart Rate Wearables and the Implications for Psychophysiological Research
Source: Psychophysiology. 2025 Feb 4;62(2):e70004. doi: 10.1111/psyp.70004 (PMC11794680; doi:10.1111/psyp.70004)
Supplement: Supplementary file 1 — Appendix S1. [file PSYP-62-e70004-s001.docx]

**Quality in Question - Supplementary Materials**

# Method

## Participants

| **Device/Condition** | **Kyto** | **HeartMath** | **Rhythm** | **Empatica** |
| --- | --- | --- | --- | --- |
| **Sitting** | 30 | 40 | 36 | 34 |
| **Arithmetic** | 32 | 40 | 36 | 34 |
| **Recovery** | 31 | 40 | 36 | 34 |
| **Standing** | 30 | 40 | 36 | 34 |
| **Breathing** | 32 | 40 | 36 | 34 |
| **Neurotask** | 29 | 40 | 36 | 34 |
| **Walking** | 32 | 40 | 36 | 32 |
| **Biking** | 29 | 40 | 36 | 31 |

Supplementary Table 1 - Number of Included Participants in the Analysis: Each row represents a condition, and each column shows a device. The number indicates the count of included participants for a given condition and device across all three analyses: mean absolute percentage error, regression analysis, and Bland-Altman analysis.

## Data Processing

### Pre-processing

Figure 1 illustrates the time series for one participant (P37) during the breathing condition, where heart rate variability is high. The upper panel shows the raw ECG in the VU-DAMS software, clearly indicating no ectopic beats or outliers. Four beats are colored yellow, suggesting their potential as outliers due to the participant’s high HRV, influenced by respiratory sinus arrhythmia. Upon extracting the the inter-beat intervals (IBI) and applying the Karlsson pre-processing approach with a custom removing threshold of 0.25, these “potential outliers” are immediately identified as actual outliers, removed, and interpolated. The red lines represent the data before pre-processing, and the black lines represent the data after pre-processing. Adjusting the custom removing threshold to 0.30 addresses this issue, ensuring that no IBI in the criterion ECG are mistakenly identified as outliers, while still allowing the detection and correction of actual outliers in other devices (HeartMath, in this example) where outliers are effectively identified after this adjustment. This modification minimizes overcorrection in individuals with inherently higher heart rate variability.


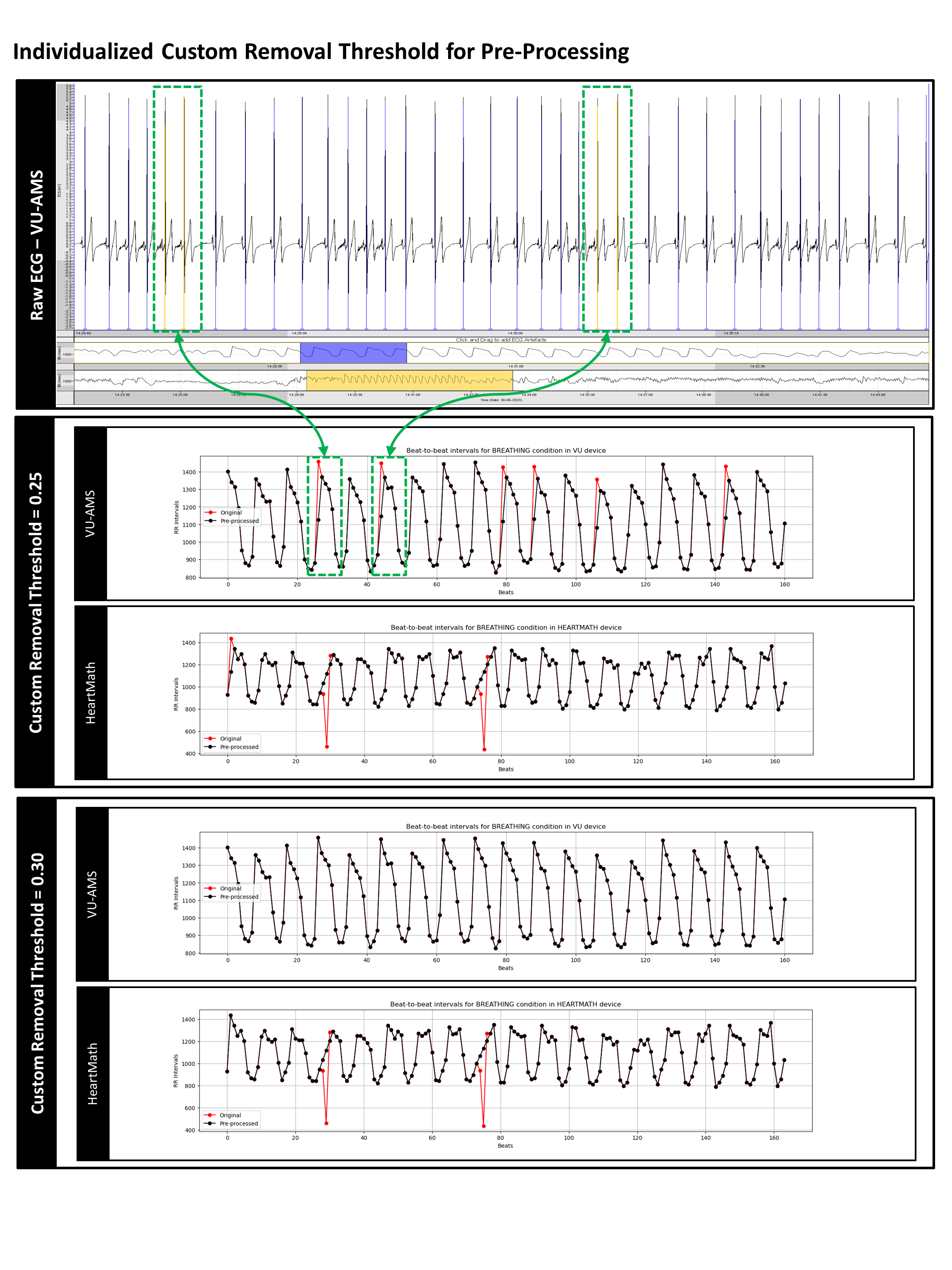


Supplementary Figure 1 - Individualized Pre-Processing Approach: This figure illustrates the effect of pre-processing on interbeat intervals. The red lines represent the interbeat intervals before pre-processing, and the black lines show them after pre-processing. Where the line is red, it indicates that the IBI has been detected as an outlier, subsequently removed, and interpolated. Although the raw ECG shows no actual ectopic beats, motion artifacts, or outliers, the pre-processing using the Karlsson method with a custom threshold of 0.25 incorrectly identifies normal IBIs in the criterion device (VU-AMS) as outliers. Adjusting this threshold to 0.30 fixes this overcorrection issue, while still enabling effective pre-processing of data from other devices (HeartMath in this example, in which actual outliers are present and correctly detected).


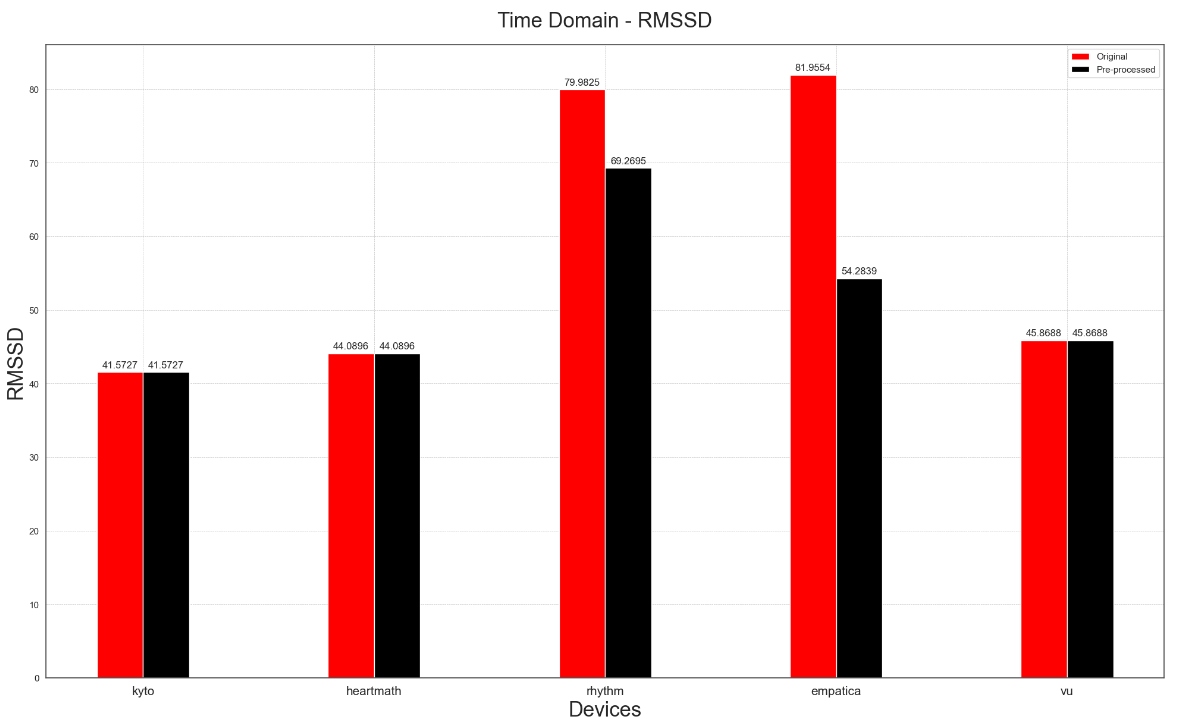


Supplementary Figure 2 - The Effect of Pre-Processing on Different Devices: This figure demonstrates how the pre-processing approach for one participant (P30) in the recovery condition helped to align the values of each device more closely with the actual values as measured by the criterion device (VU-AMS). The red bars represent the data before pre-processing, and the black bars show the data after pre-processing.

## VU-AMS Acceleration

The VU-AMS is equipped with a tri-axial accelerometer. All participants wore the VU-AMS on the left hip using a belt to ensure accurate accelerometer measurements. We extracted the motility signal (with units in g), which is based on the y-axis of the tri-axial accelerometer with a resolution of 1 Hz. This was exported as an ASCII file using VU-DAMS software. For each participant, we then segmented this continuous signal into the experimental conditions. For each condition, we calculated the root mean square (RMS) of the signal using the following equation:

$$RMS=\sqrt{\frac{1}{n}\sum_{i=1}^{n} {acc}_{i}^{2}}$$

Where ${acc}_{i}$ represents the $i^{th}$ acceleration measurement, and $n$ is the total number of measurements in a condition.

# Results

## Signal Quality

The Table 2 provides the underlying data used to create Figures 2 in the manuscript. Each value represents the number of participants. For example, in the sitting condition for Kyto, out of 40 participants, the signal was acceptable for 28 people, poor for 2, and for 10 people the device did not work properly, resulting in missing data.

| **Conditions / Devices** | **Signal Quality** | **Kyto** | **Rhythm** | **HeartMath** | **Empatica** |
| --- | --- | --- | --- | --- | --- |
| **Sitting** | Acceptable | 28 | 30 | 40 | 34 |
|  | Poor | 2 | 6 | 0 | 0 |
|  | Missing | 10 | 4 | 0 | 0 |
| **Recovery** | Acceptable | 29 | 28 | 40 | 33 |
|  | Poor | 2 | 8 | 0 | 1 |
|  | Missing | 9 | 4 | 0 | 0 |
| **Breathing** | Acceptable | 26 | 25 | 40 | 34 |
|  | Poor | 6 | 11 | 0 | 0 |
|  | Missing | 8 | 4 | 0 | 0 |
| **Neurotask** | Acceptable | 26 | 32 | 40 | 32 |
|  | Poor | 3 | 4 | 0 | 2 |
|  | Missing | 11 | 4 | 0 | 0 |
| **Arithmetic** | Acceptable | 28 | 14 | 40 | 23 |
|  | Poor | 3 | 22 | 0 | 11 |
|  | Missing | 9 | 4 | 0 | 0 |
| **Standing** | Acceptable | 24 | 26 | 40 | 30 |
|  | Poor | 5 | 10 | 0 | 4 |
|  | Missing | 11 | 4 | 0 | 0 |
| **Walking** | Acceptable | 14 | 5 | 40 | 6 |
|  | Poor | 18 | 31 | 0 | 26 |
|  | Missing | 8 | 4 | 0 | 2 |
| **Biking** | Acceptable | 15 | 11 | 24 | 7 |
|  | Poor | 14 | 25 | 16 | 24 |
|  | Missing | 11 | 4 | 0 | 3 |

Supplementary Table 2 - Signal Quality Report for All Devices: Signal quality is labeled as “poor” if either the number of detected IBIs in a given device under specific conditions differs by more than 30 percent compared to the counterpart in the criterion device, or if more than 30 percent of the detected IBIs in a device under a condition contain artifacts. “Missing” refers to instances where data was not recorded due to device malfunction. Each value represents the number of participants. The total number of participants for whom we attempted to include Kyto, Rhythm and HearthMath wearables was N=40, and for Empatica it was N=34 (six participants did not wear the Empatica).

## Descriptive Statistics

Descriptive statistics of RMSSD, HF, and mean HR for all devices in all conditions.

|  | **Feature** | **RMSSD** | | | | **HF Component** | | | | | **Mean Heart Rate** | | | |
| --- | --- | --- | --- | --- | --- | --- | --- | --- | --- | --- | --- | --- | --- | --- |
|  |  | **max** | **mean** | **min** | **std** | **max** | **mean** | **min** | **std** | **max** | | **mean** | **min** | **std** |
| **Device** | **Condition** |  |  |  |  |  |  |  |  |  | |  |  |  |
| **kyto** | **sitting** | 78.39 | 32.85 | 7.71 | 17.94 | 4490.83 | 770.19 | 56.69 | 957.16 | 96.78 | | 71.04 | 54.27 | 9.21 |
|  | **arithmetic** | 100.4 | 44.01 | 10.5 | 22.57 | 4267.18 | 1229.85 | 102.77 | 1124.46 | 112.01 | | 81.2 | 59.79 | 11.9 |
|  | **recovery** | 100.54 | 35.34 | 9.06 | 18.67 | 7352.27 | 892.14 | 68.67 | 1436.18 | 94.02 | | 70.62 | 56.12 | 8.44 |
|  | **standing** | 67.54 | 30.19 | 5.78 | 16.47 | 3560.61 | 637.59 | 29.43 | 845.45 | 106.97 | | 79.04 | 60.33 | 10.12 |
|  | **breathing** | 137.1 | 62.09 | 18.49 | 28.9 | 11351.92 | 1619.15 | 82.81 | 2163.14 | 95.34 | | 67.63 | 55.63 | 8.38 |
|  | **neurotask** | 76.15 | 35.06 | 11.57 | 17.74 | 3446.89 | 681.03 | 65.45 | 737.48 | 90.26 | | 70.22 | 52.07 | 9.85 |
|  | **walking** | 222.23 | 78.76 | 17.16 | 57.91 | 8629 | 2918.33 | 80.92 | 2944.36 | 98.08 | | 77.22 | 43.85 | 13.2 |
|  | **biking** | 134.46 | 48.95 | 5.45 | 37.7 | 8509.4 | 1610.74 | 5.01 | 2132.01 | 144.78 | | 107.21 | 66.62 | 20.3 |
| **rhythm** | **sitting** | 170.3 | 61.39 | 18.85 | 35.97 | 9240.98 | 1803.83 | 85.23 | 2058.79 | 94.82 | | 73.58 | 52.91 | 9.83 |
|  | **arithmetic** | 161.16 | 76.78 | 30.11 | 35.05 | 10912.4 | 2535.96 | 314.83 | 2605.89 | 110.08 | | 81.18 | 56.81 | 12.8 |
|  | **recovery** | 152.69 | 62.01 | 16.32 | 34.24 | 7090.81 | 1641.69 | 103.54 | 2039.36 | 92.27 | | 72.26 | 54.08 | 9.54 |
|  | **standing** | 140.94 | 54.87 | 19.37 | 29.59 | 8469 | 1226.6 | 55.84 | 1858.21 | 104.93 | | 80.98 | 59.52 | 10.9 |
|  | **breathing** | 179.91 | 88.73 | 39.65 | 32.33 | 11271.14 | 3330.92 | 450.99 | 2281.49 | 94.24 | | 70.37 | 53.52 | 9.68 |
|  | **neurotask** | 159.28 | 60.23 | 23.23 | 32.02 | 7373.54 | 1341.14 | 107.9 | 1824.03 | 89.15 | | 72.35 | 51.08 | 9.93 |
|  | **walking** | 159.61 | 96.36 | 29.53 | 28.54 | 9851.03 | 3367.6 | 261.61 | 2254.96 | 154.83 | | 87.89 | 68.32 | 15.05 |
|  | **biking** | 121.21 | 58.64 | 22.9 | 23.87 | 6706.16 | 1123.1 | 78.47 | 1486.49 | 158.84 | | 119.44 | 90.9 | 21.07 |
| **heartmath** | **sitting** | 122.39 | 47.15 | 17.11 | 24.41 | 6310.76 | 1364.88 | 110.16 | 1582.21 | 97.25 | | 74.09 | 54.88 | 9.81 |
|  | **arithmetic** | 120.81 | 57.12 | 22.94 | 20.36 | 5028.31 | 1400.95 | 120.53 | 1198.15 | 112.57 | | 84.36 | 60.4 | 13.08 |
|  | **recovery** | 111.62 | 50.38 | 14.17 | 23.63 | 7012.42 | 1256.06 | 89.75 | 1502.59 | 94.45 | | 72.78 | 55.52 | 9.79 |
|  | **standing** | 64.84 | 36.67 | 11.91 | 12.73 | 3053.61 | 636.3 | 32.32 | 693.8 | 107.36 | | 82.3 | 60.71 | 11.19 |
|  | **breathing** | 175.45 | 72.38 | 22.24 | 33.4 | 9023.58 | 1777.49 | 227.41 | 1714.35 | 95.75 | | 70.95 | 56.35 | 9.28 |
|  | **neurotask** | 101.1 | 49.43 | 19.37 | 20.38 | 3831.62 | 970.04 | 104.2 | 950.64 | 91.7 | | 72.54 | 52.7 | 10.07 |
|  | **walking** | 87.91 | 46.64 | 14.23 | 15.06 | 2144.64 | 485.39 | 25.29 | 398.18 | 108.34 | | 85.1 | 57.63 | 11.15 |
|  | **biking** | 140.45 | 52.33 | 11.13 | 27.44 | 6091.38 | 1387.49 | 9.27 | 1687.91 | 145.02 | | 117.9 | 87 | 13.87 |
| **empatica** | **sitting** | 162.54 | 60.49 | 10.84 | 32.32 | 8060.94 | 1725.2 | 71.73 | 2076.92 | 94.81 | | 70.88 | 53.62 | 9.44 |
|  | **arithmetic** | 203.9 | 101.86 | 26.6 | 36.44 | 12727.88 | 4499.09 | 282.41 | 3285.68 | 92.01 | | 75.44 | 59.19 | 8.42 |
|  | **recovery** | 193.28 | 72.29 | 12.88 | 36.54 | 11382.97 | 2181.3 | 92.26 | 2882.27 | 92.17 | | 69.73 | 54.69 | 8.83 |
|  | **standing** | 137.22 | 61.7 | 10.94 | 28.48 | 10663.32 | 1959.51 | 37.93 | 2570.38 | 104.65 | | 78.09 | 59.26 | 10.1 |
|  | **breathing** | 205.9 | 89.13 | 28.54 | 39.3 | 13806.66 | 2173.29 | 264.68 | 2475.82 | 93.41 | | 68.17 | 55.33 | 8.56 |
|  | **neurotask** | 194.6 | 77.77 | 22.08 | 41.99 | 10015.82 | 1930.31 | 142.32 | 2318.67 | 88.45 | | 69.26 | 51.1 | 9.59 |
|  | **walking** | 186.18 | 117.92 | 57.85 | 28.57 | 15027.15 | 5763.64 | 1626.7 | 3355.08 | 92.71 | | 77.3 | 69.09 | 6.09 |
|  | **biking** | 158.44 | 87.29 | 33.13 | 27.54 | 12373.06 | 3540.55 | 699.88 | 2390.57 | 126.48 | | 91.15 | 62.83 | 11.7 |
| **Vu**  **(Criterion)** | **sitting** | 110.01 | 43.54 | 9.26 | 25.58 | 7208.87 | 1299.64 | 56.06 | 1654.52 | 94.8 | | 72.23 | 53.5 | 9.7 |
|  | **arithmetic** | 103.42 | 41.28 | 11.93 | 20.19 | 5470.79 | 1163.76 | 101.3 | 1139.82 | 109.64 | | 82.29 | 58.57 | 13.1 |
|  | **recovery** | 112.71 | 46.7 | 10.44 | 24.06 | 8459.1 | 1293.6 | 74.61 | 1793.06 | 92.17 | | 71.05 | 54.21 | 9.54 |
|  | **standing** | 59.15 | 28.43 | 5.84 | 13.99 | 2921.46 | 563.72 | 22.97 | 656.48 | 104.66 | | 80.33 | 59.24 | 10.94 |
|  | **breathing** | 199.58 | 75.04 | 23.63 | 38.83 | 11592.41 | 1696.96 | 260.8 | 1954.03 | 93.42 | | 69.04 | 54.75 | 8.94 |
|  | **neurotask** | 114.43 | 47.64 | 16.91 | 24.89 | 4605.07 | 920.17 | 79.24 | 1077.04 | 89.44 | | 70.69 | 51.37 | 9.87 |
|  | **walking** | 90.16 | 29.9 | 10 | 15.06 | 2354.67 | 368.92 | 21 | 429 | 105.87 | | 82.95 | 55.97 | 10.83 |
|  | **biking** | 28.19 | 6.7 | 1.56 | 5.17 | 872.8 | 39.76 | 0.24 | 137.73 | 157.7 | | 127.93 | 89.84 | 18.57 |

Supplementary Table 3 - Descriptive Statistics of RMSSD, HF, and Mean HR for All Devices in All Conditions: This table displays the mean, standard deviation (std), maximum value (max), and minimum value (min) for the RMSSD, HF component, and mean HR across all devices and conditions. RMSSD: root mean square of successive differences; HF: high frequency component; Mean HR: mean heart rate.

## VU-AMS Acceleration

Figure 3 illustrates the movement associated with each condition, based on the motility signal from VU-AMS. The majority of movements were observed in the biking and walking conditions. The other conditions showed similar activity levels, likely due to the accelerometer worn on the left hip, which is less sensitive to subtle body movements, thereby not differentiating between these conditions.


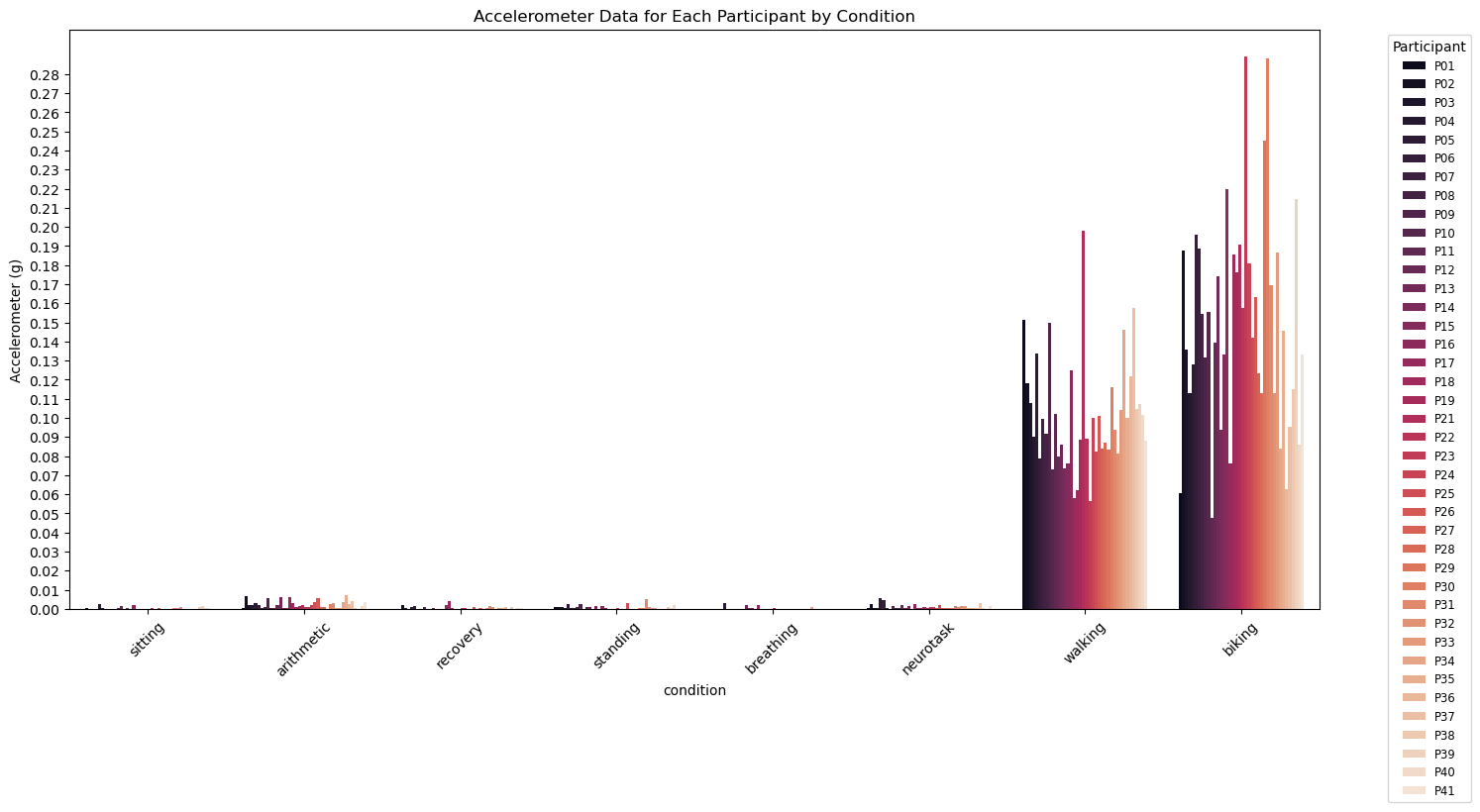


Supplementary Figure 3 - Acceleration Data: The x-axis displays different experimental conditions. The y-axis represents acceleration (in g) based on the Y-axis of the tri-axial accelerometer (motility signal). For each condition, the root mean square (RMS) of the signal has been calculated. Each color in the graph represents a single participant.

## Mean Arctangent Absolute Percentage Error (MAAPE)

| **Device** | **Feature** | **Condition** | **MAAPE** | **CI Lower** | **CI Upper** |
| --- | --- | --- | --- | --- | --- |
| kyto | RMSSD | sitting | 22.78 | 19.56 | 26.01 |
| kyto | RMSSD | arithmetic | 25.39 | 14.69 | 36.08 |
| kyto | RMSSD | recovery | 25.05 | 19.53 | 30.56 |
| kyto | RMSSD | standing | 21.96 | 13.73 | 30.19 |
| kyto | RMSSD | breathing | 17.51 | 13.76 | 21.26 |
| kyto | RMSSD | neurotask | 28.66 | 25.24 | 32.08 |
| kyto | RMSSD | walking | 74.41 | 55.46 | 93.36 |
| kyto | RMSSD | biking | 118.12 | 102.82 | 133.43 |
| kyto | HF | sitting | 27.35 | 21.6 | 33.09 |
| kyto | HF | arithmetic | 35.65 | 22.82 | 48.48 |
| kyto | HF | recovery | 32.95 | 23.83 | 42.06 |
| kyto | HF | standing | 40.26 | 25.86 | 54.67 |
| kyto | HF | breathing | 32.18 | 23.35 | 41 |
| kyto | HF | neurotask | 27.82 | 22.33 | 33.31 |
| kyto | HF | walking | 101.65 | 80.6 | 122.7 |
| kyto | HF | biking | 127.87 | 111.21 | 144.53 |
| kyto | Mean HR | sitting | 1.67 | 1.53 | 1.8 |
| kyto | Mean HR | arithmetic | 2.14 | 1.36 | 2.92 |
| kyto | Mean HR | recovery | 1.68 | 1.51 | 1.85 |
| kyto | Mean HR | standing | 2.44 | 0.96 | 3.92 |
| kyto | Mean HR | breathing | 2.25 | 1.63 | 2.87 |
| kyto | Mean HR | neurotask | 1.46 | 1.26 | 1.67 |
| kyto | Mean HR | walking | 6.72 | 3.1 | 10.34 |
| kyto | Mean HR | biking | 13.89 | 8.24 | 19.53 |
| rhythm | RMSSD | sitting | 41.62 | 31.39 | 51.85 |
| rhythm | RMSSD | arithmetic | 68.09 | 55.27 | 80.91 |
| rhythm | RMSSD | recovery | 35.5 | 24.54 | 46.45 |
| rhythm | RMSSD | standing | 73.62 | 61.6 | 85.63 |
| rhythm | RMSSD | breathing | 29.08 | 20.49 | 37.67 |
| rhythm | RMSSD | neurotask | 34.56 | 23.95 | 45.17 |
| rhythm | RMSSD | walking | 110.16 | 98.97 | 121.35 |
| rhythm | RMSSD | biking | 140.21 | 133.92 | 146.49 |
| rhythm | HF | sitting | 52.08 | 39.87 | 64.29 |
| rhythm | HF | arithmetic | 79.04 | 65.31 | 92.76 |
| rhythm | HF | recovery | 42.22 | 29.93 | 54.5 |
| rhythm | HF | standing | 81.21 | 66.95 | 95.48 |
| rhythm | HF | breathing | 81.68 | 66.05 | 97.31 |
| rhythm | HF | neurotask | 49.16 | 34.57 | 63.76 |
| rhythm | HF | walking | 138.18 | 128.93 | 147.43 |
| rhythm | HF | biking | 146.94 | 137.84 | 156.05 |
| rhythm | Mean HR | sitting | 2.39 | -0.22 | 5 |
| rhythm | Mean HR | arithmetic | 3.07 | 1.03 | 5.11 |
| rhythm | Mean HR | recovery | 2.59 | -0.1 | 5.28 |
| rhythm | Mean HR | standing | 1.52 | 0.19 | 2.85 |
| rhythm | Mean HR | breathing | 3.94 | 0.4 | 7.49 |
| rhythm | Mean HR | neurotask | 2.92 | -0.64 | 6.47 |
| rhythm | Mean HR | walking | 11.01 | 6.61 | 15.4 |
| rhythm | Mean HR | biking | 6.85 | 3.44 | 10.26 |
| heartmath | RMSSD | sitting | 17.03 | 11.33 | 22.73 |
| heartmath | RMSSD | arithmetic | 40.93 | 28.9 | 52.96 |
| heartmath | RMSSD | recovery | 17.07 | 12.3 | 21.84 |
| heartmath | RMSSD | standing | 35.94 | 24.99 | 46.88 |
| heartmath | RMSSD | breathing | 7.7 | 5.21 | 10.19 |
| heartmath | RMSSD | neurotask | 16.65 | 11.2 | 22.1 |
| heartmath | RMSSD | walking | 52.75 | 39.59 | 65.92 |
| heartmath | RMSSD | biking | 129.09 | 117.62 | 140.56 |
| heartmath | HF | sitting | 23.58 | 15.89 | 31.27 |
| heartmath | HF | arithmetic | 28.93 | 17.57 | 40.29 |
| heartmath | HF | recovery | 15.38 | 11.89 | 18.87 |
| heartmath | HF | standing | 32.02 | 21.02 | 43.03 |
| heartmath | HF | breathing | 15.99 | 10.15 | 21.83 |
| heartmath | HF | neurotask | 22.48 | 15.29 | 29.67 |
| heartmath | HF | walking | 46.4 | 33.43 | 59.37 |
| heartmath | HF | biking | 125.88 | 110.6 | 141.16 |
| heartmath | Mean HR | sitting | 2.6 | 2.34 | 2.86 |
| heartmath | Mean HR | arithmetic | 2.58 | 2.18 | 2.99 |
| heartmath | Mean HR | recovery | 2.44 | 2.31 | 2.56 |
| heartmath | Mean HR | standing | 2.46 | 2.42 | 2.5 |
| heartmath | Mean HR | breathing | 2.75 | 2.34 | 3.16 |
| heartmath | Mean HR | neurotask | 2.63 | 2.41 | 2.85 |
| heartmath | Mean HR | walking | 2.59 | 2.46 | 2.73 |
| heartmath | Mean HR | biking | 8.91 | 5.35 | 12.46 |
| empatica | RMSSD | sitting | 36.16 | 23.7 | 48.62 |
| empatica | RMSSD | arithmetic | 87.98 | 73.79 | 102.17 |
| empatica | RMSSD | recovery | 48.62 | 35.4 | 61.85 |
| empatica | RMSSD | standing | 74.01 | 58.83 | 89.19 |
| empatica | RMSSD | breathing | 20.98 | 11.72 | 30.23 |
| empatica | RMSSD | neurotask | 52.92 | 38.41 | 67.44 |
| empatica | RMSSD | walking | 122.58 | 115.73 | 129.44 |
| empatica | RMSSD | biking | 148.68 | 146.61 | 150.76 |
| empatica | HF | sitting | 30.57 | 17.86 | 43.27 |
| empatica | HF | arithmetic | 100.72 | 82.03 | 119.41 |
| empatica | HF | recovery | 51.34 | 35.05 | 67.62 |
| empatica | HF | standing | 77.56 | 56.9 | 98.23 |
| empatica | HF | breathing | 33.55 | 19.6 | 47.49 |
| empatica | HF | neurotask | 69.55 | 50.81 | 88.29 |
| empatica | HF | walking | 150.3 | 148.15 | 152.44 |
| empatica | HF | biking | 156.35 | 155.89 | 156.8 |
| empatica | Mean HR | sitting | 0.49 | -0.09 | 1.06 |
| empatica | Mean HR | arithmetic | 6.6 | 3.74 | 9.46 |
| empatica | Mean HR | recovery | 1.09 | 0.36 | 1.81 |
| empatica | Mean HR | standing | 1.58 | 0.28 | 2.88 |
| empatica | Mean HR | breathing | 0.8 | 0.28 | 1.33 |
| empatica | Mean HR | neurotask | 1.01 | 0.35 | 1.67 |
| empatica | Mean HR | walking | 9.22 | 6.37 | 12.07 |
| empatica | Mean HR | biking | 26.83 | 21.96 | 31.69 |

Supplementary Table 4 - Mean Arctangent Absolute Percentage Error (MAAPE): values representing MAAPE are listed for each condition and feature, together with the 95% CI, in all the devices. RMSSD: root mean square of successive differences; HF: high frequency component; Mean HR: mean heart rate.

## Regression Analysis

Figure 4, Figure 5, and Figure 6 display scatter plots for LnRMSSD (log-transformed RMSSD), the LnHF (log-transformed HF) component, and mean HR of the devices. Each plot is presented for each condition, plotted against the corresponding values of the criterion device. The y-axis in these plots represents the values from the criterion device.


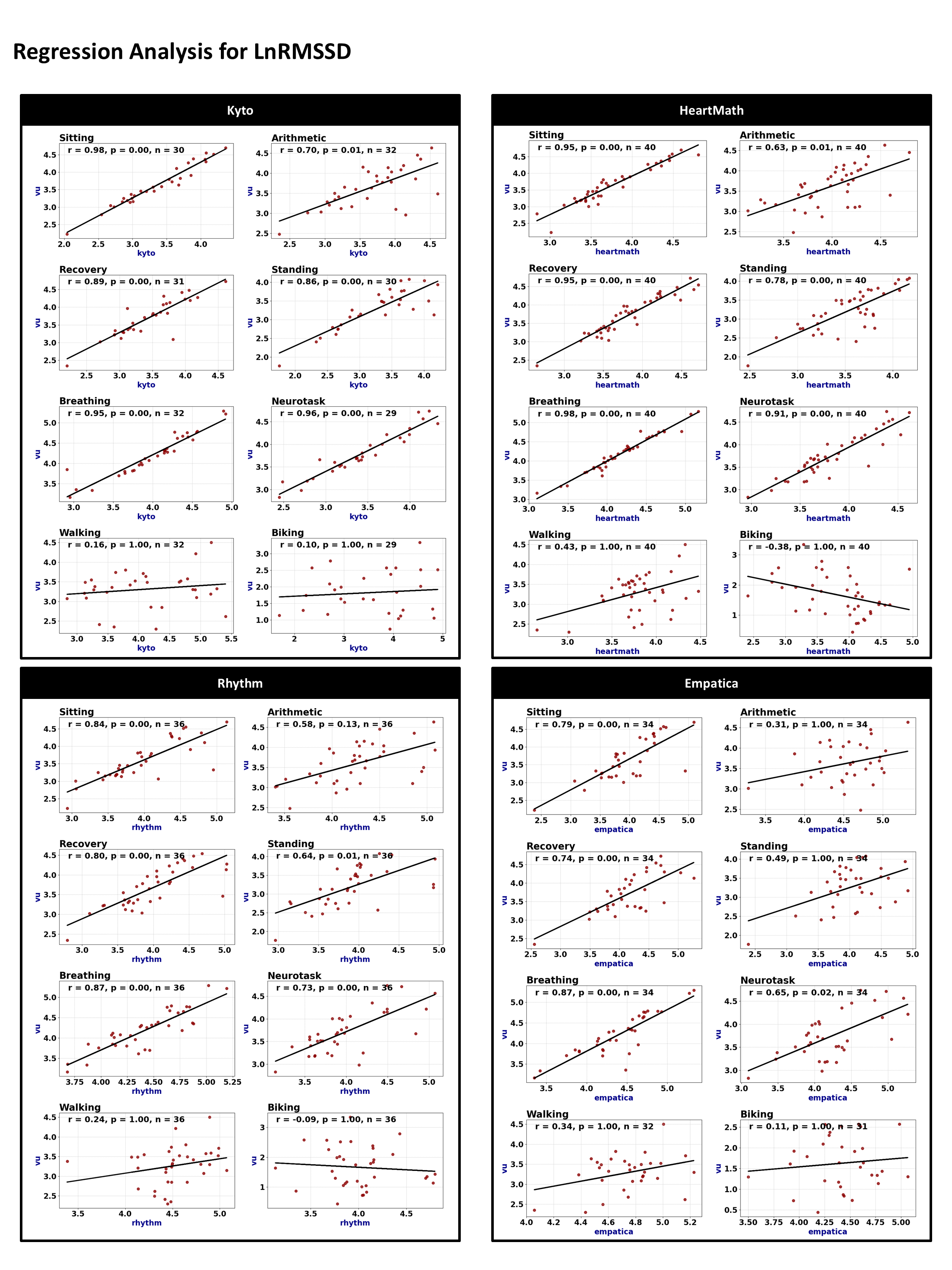


Supplementary Figure 4 - Scatter Plot of LnRMSSD (log-transformed RMSSD) for Wearables vs. Criterion Device: Each subplot shows an experimental condition, with 'r' as Pearson correlation, 'p' as Bonferroni-adjusted significance, and 'n' as observations count. The y-axis is the criterion device (VU-AMS) values, and the x-axis shows each wearable's values, all in log-transformed units.


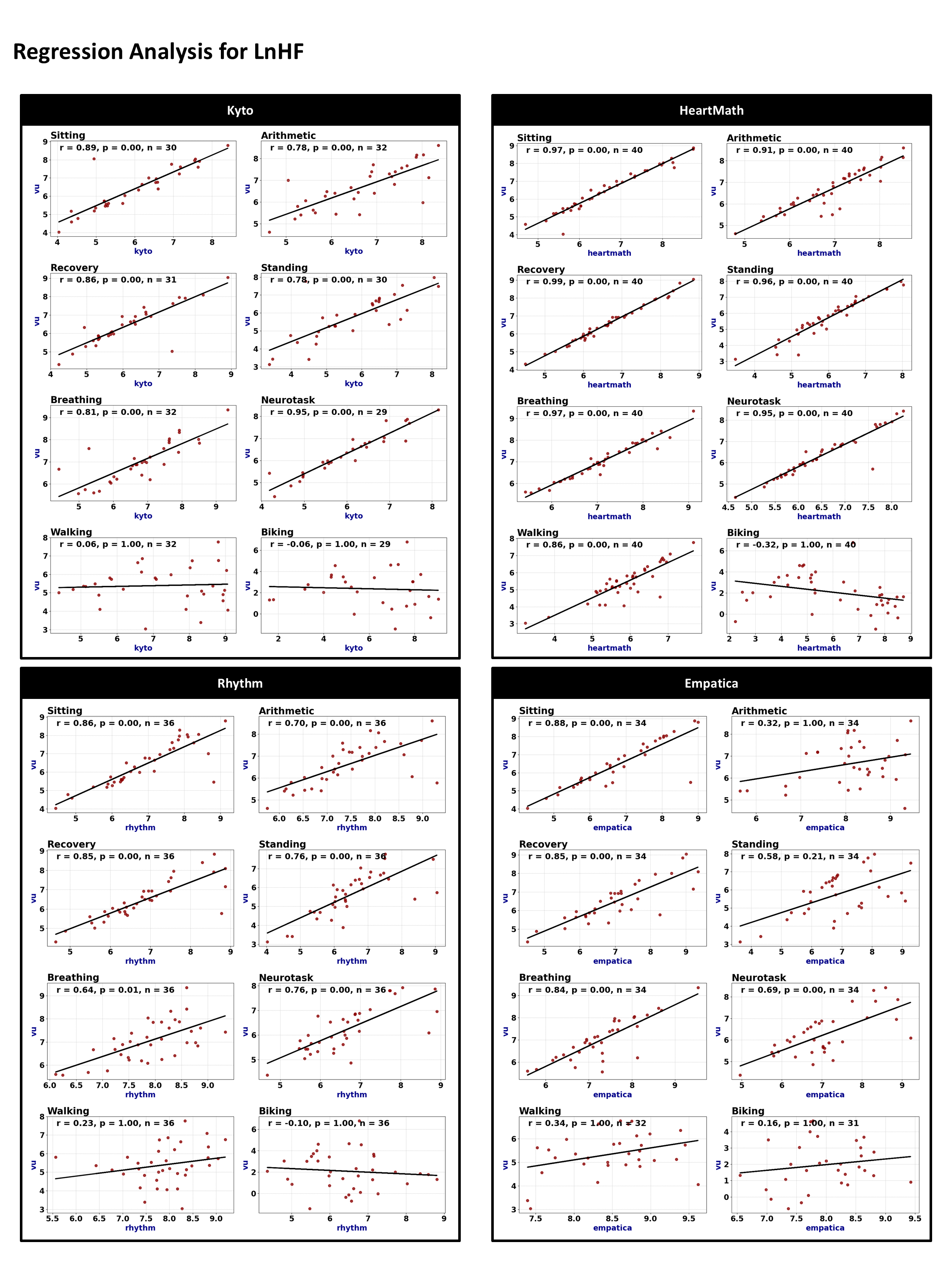


Supplementary Figure 5 - Scatter Plot of LnHF for Wearables vs. Criterion Device: Each subplot shows an experimental condition, with 'r' as Pearson correlation, 'p' as Bonferroni-adjusted significance, and 'n' as observations count. The y-axis is the criterion device (VU-AMS) values, and the x-axis shows each wearable's values, all in log-transformed units.


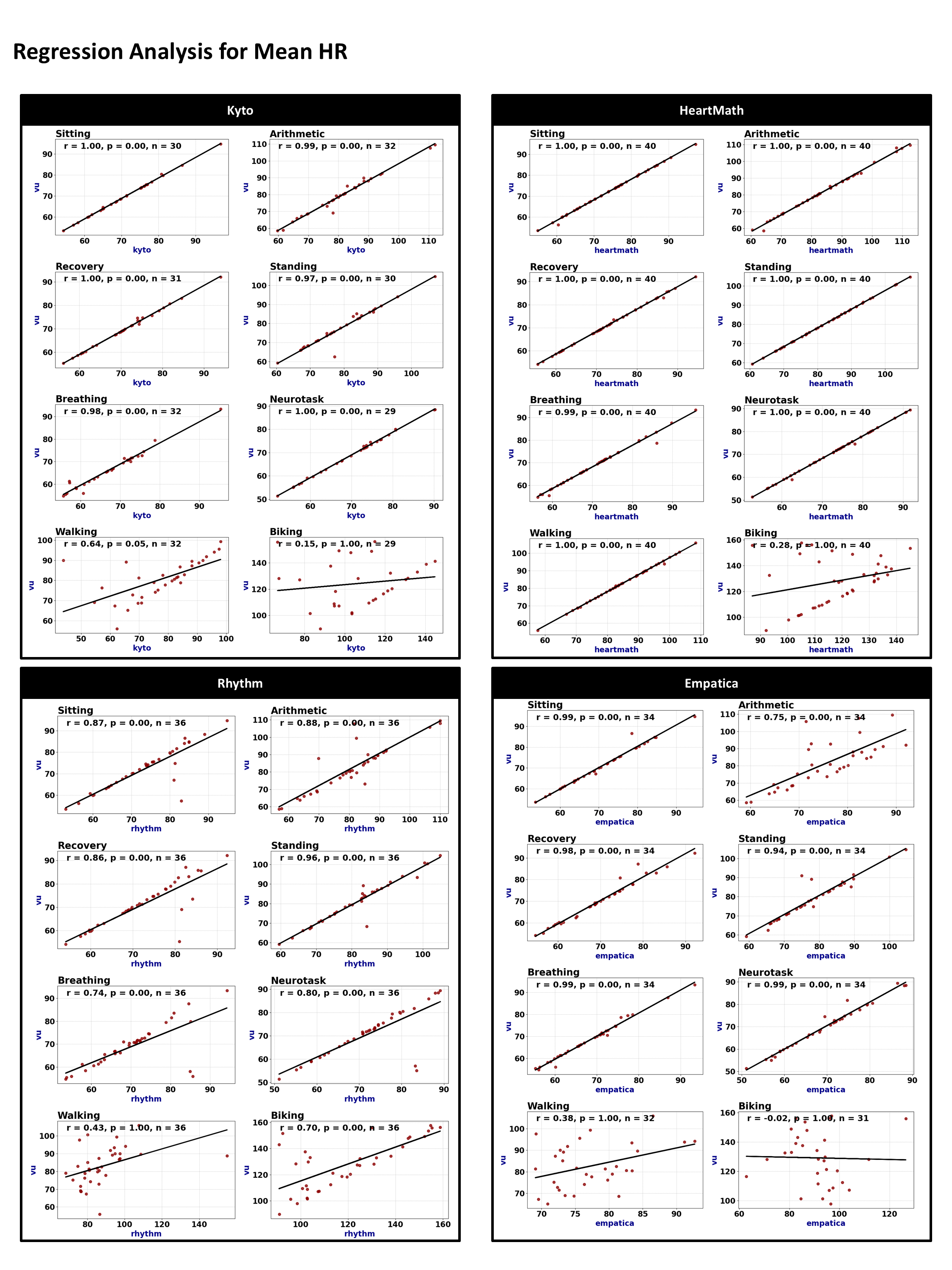


Supplementary Figure 6 - Scatter Plot of Mean HR for Wearables vs. Criterion Device: Each subplot shows an experimental condition, with 'r' as Pearson correlation, 'p' as Bonferroni-adjusted significance, and 'n' as observations count. The y-axis is the criterion device values, and the x-axis shows each wearable's values.

## Bland-Altman Analysis

| **Device** | **Feature** | **Condition** | **Bias with 95% CI** | **SD** | **Lower LOA with 95% CI** | **Upper LOA with 95% CI** |
| --- | --- | --- | --- | --- | --- | --- |
| kyto | LnRMSSD | sitting | -0.27 [-0.32, -0.22] | 0.13 | -0.52 [-0.56, -0.47] | -0.02 [-0.07, 0.02] |
| kyto | LnRMSSD | arithmetic | 0.01 [-0.14, 0.16] | 0.41 | -0.78 [-0.93, -0.64] | 0.8 [0.66, 0.95] |
| kyto | LnRMSSD | recovery | -0.25 [-0.34, -0.16] | 0.25 | -0.74 [-0.82, -0.65] | 0.23 [0.14, 0.32] |
| kyto | LnRMSSD | standing | -0.02 [-0.14, 0.09] | 0.31 | -0.63 [-0.74, -0.52] | 0.58 [0.47, 0.69] |
| kyto | LnRMSSD | breathing | -0.21 [-0.27, -0.15] | 0.17 | -0.54 [-0.6, -0.48] | 0.12 [0.06, 0.18] |
| kyto | LnRMSSD | neurotask | -0.36 [-0.42, -0.31] | 0.15 | -0.65 [-0.71, -0.6] | -0.07 [-0.13, -0.02] |
| kyto | LnRMSSD | walking | 0.79 [0.49, 1.09] | 0.82 | -0.82 [-1.11, -0.54] | 2.4 [2.12, 2.69] |
| kyto | LnRMSSD | biking | 1.73 [1.33, 2.13] | 1.05 | -0.33 [-0.71, 0.05] | 3.79 [3.41, 4.17] |
| kyto | LnHF | sitting | -0.4 [-0.61, -0.19] | 0.56 | -1.5 [-1.7, -1.3] | 0.71 [0.5, 0.91] |
| kyto | LnHF | arithmetic | -0.01 [-0.26, 0.23] | 0.68 | -1.34 [-1.58, -1.11] | 1.32 [1.08, 1.55] |
| kyto | LnHF | recovery | -0.3 [-0.51, -0.09] | 0.56 | -1.41 [-1.61, -1.21] | 0.81 [0.61, 1.01] |
| kyto | LnHF | standing | -0.02 [-0.34, 0.31] | 0.86 | -1.71 [-2.01, -1.4] | 1.67 [1.37, 1.98] |
| kyto | LnHF | breathing | -0.22 [-0.45, 0.02] | 0.65 | -1.5 [-1.73, -1.27] | 1.06 [0.84, 1.29] |
| kyto | LnHF | neurotask | -0.31 [-0.43, -0.19] | 0.32 | -0.93 [-1.05, -0.82] | 0.31 [0.19, 0.42] |
| kyto | LnHF | walking | 1.8 [1.17, 2.43] | 1.75 | -1.63 [-2.23, -1.02] | 5.23 [4.62, 5.84] |
| kyto | LnHF | biking | 3.71 [2.65, 4.77] | 2.79 | -1.76 [-2.77, -0.74] | 9.17 [8.16, 10.19] |
| kyto | Mean HR | sitting | 1.17 [1.05, 1.3] | 0.33 | 0.52 [0.4, 0.64] | 1.83 [1.71, 1.95] |
| kyto | Mean HR | arithmetic | 1.43 [0.81, 2.06] | 1.74 | -1.97 [-2.58, -1.37] | 4.84 [4.24, 5.44] |
| kyto | Mean HR | recovery | 1.12 [0.94, 1.3] | 0.49 | 0.15 [-0.02, 0.33] | 2.09 [1.92, 2.26] |
| kyto | Mean HR | standing | 1.65 [0.67, 2.62] | 2.61 | -3.47 [-4.4, -2.54] | 6.76 [5.83, 7.7] |
| kyto | Mean HR | breathing | 0.93 [0.37, 1.49] | 1.55 | -2.11 [-2.64, -1.57] | 3.97 [3.43, 4.51] |
| kyto | Mean HR | neurotask | 1.02 [0.85, 1.19] | 0.44 | 0.15 [-0.01, 0.32] | 1.89 [1.73, 2.05] |
| kyto | Mean HR | walking | -3.21 [-6.92, 0.5] | 10.29 | -23.37 [-26.94, -19.81] | 16.95 [13.39, 20.52] |
| kyto | Mean HR | biking | -17.15 [-26.62, -7.68] | 24.9 | -65.95 [-75.01, -56.89] | 31.66 [22.59, 40.72] |
| rhythm | LnRMSSD | sitting | 0.38 [0.28, 0.49] | 0.32 | -0.24 [-0.35, -0.14] | 1.01 [0.91, 1.12] |
| rhythm | LnRMSSD | arithmetic | 0.66 [0.51, 0.81] | 0.43 | -0.19 [-0.33, -0.05] | 1.51 [1.37, 1.65] |
| rhythm | LnRMSSD | recovery | 0.32 [0.21, 0.43] | 0.33 | -0.32 [-0.43, -0.21] | 0.96 [0.85, 1.07] |
| rhythm | LnRMSSD | standing | 0.72 [0.57, 0.86] | 0.43 | -0.12 [-0.26, 0.02] | 1.55 [1.41, 1.69] |
| rhythm | LnRMSSD | breathing | 0.23 [0.14, 0.32] | 0.26 | -0.27 [-0.35, -0.19] | 0.73 [0.65, 0.81] |
| rhythm | LnRMSSD | neurotask | 0.28 [0.17, 0.4] | 0.34 | -0.39 [-0.5, -0.27] | 0.95 [0.84, 1.06] |
| rhythm | LnRMSSD | walking | 1.25 [1.08, 1.43] | 0.52 | 0.24 [0.07, 0.41] | 2.27 [2.1, 2.43] |
| rhythm | LnRMSSD | biking | 2.34 [2.07, 2.61] | 0.81 | 0.76 [0.49, 1.02] | 3.92 [3.66, 4.19] |
| rhythm | LnHF | sitting | 0.5 [0.29, 0.71] | 0.61 | -0.7 [-0.9, -0.5] | 1.71 [1.51, 1.91] |
| rhythm | LnHF | arithmetic | 0.83 [0.58, 1.07] | 0.72 | -0.59 [-0.82, -0.35] | 2.24 [2.0, 2.48] |
| rhythm | LnHF | recovery | 0.38 [0.18, 0.58] | 0.59 | -0.77 [-0.97, -0.58] | 1.54 [1.34, 1.73] |
| rhythm | LnHF | standing | 0.87 [0.61, 1.14] | 0.78 | -0.66 [-0.92, -0.41] | 2.41 [2.15, 2.66] |
| rhythm | LnHF | breathing | 0.85 [0.61, 1.08] | 0.69 | -0.5 [-0.73, -0.28] | 2.2 [1.97, 2.42] |
| rhythm | LnHF | neurotask | 0.43 [0.2, 0.66] | 0.67 | -0.89 [-1.11, -0.67] | 1.75 [1.53, 1.97] |
| rhythm | LnHF | walking | 2.5 [2.12, 2.88] | 1.12 | 0.29 [-0.07, 0.66] | 4.7 [4.33, 5.07] |
| rhythm | LnHF | biking | 4.38 [3.66, 5.1] | 2.13 | 0.19 [-0.5, 0.89] | 8.56 [7.86, 9.26] |
| rhythm | Mean HR | sitting | 1.24 [-0.43, 2.91] | 4.94 | -8.44 [-10.05, -6.83] | 10.91 [9.3, 12.52] |
| rhythm | Mean HR | arithmetic | -1.35 [-3.5, 0.81] | 6.37 | -13.83 [-15.92, -11.75] | 11.14 [9.06, 13.22] |
| rhythm | Mean HR | recovery | 1.19 [-0.53, 2.91] | 5.08 | -8.76 [-10.42, -7.1] | 11.15 [9.49, 12.81] |
| rhythm | Mean HR | standing | 0.65 [-0.36, 1.66] | 3 | -5.22 [-6.2, -4.24] | 6.52 [5.54, 7.5] |
| rhythm | Mean HR | breathing | 1.26 [-1.04, 3.57] | 6.82 | -12.1 [-14.32, -9.87] | 14.62 [12.39, 16.85] |
| rhythm | Mean HR | neurotask | 1.47 [-0.69, 3.64] | 6.4 | -11.07 [-13.16, -8.98] | 14.01 [11.92, 16.1] |
| rhythm | Mean HR | walking | 5.06 [0.26, 9.86] | 14.19 | -22.75 [-27.38, -18.11] | 32.87 [28.23, 37.5] |
| rhythm | Mean HR | biking | -8.36 [-13.65, -3.07] | 15.64 | -39.02 [-44.14, -33.91] | 22.3 [17.19, 27.41] |
| heartmath | LnRMSSD | sitting | 0.12 [0.06, 0.18] | 0.18 | -0.24 [-0.3, -0.18] | 0.49 [0.43, 0.54] |
| heartmath | LnRMSSD | arithmetic | 0.37 [0.25, 0.5] | 0.38 | -0.37 [-0.49, -0.25] | 1.12 [1.0, 1.24] |
| heartmath | LnRMSSD | recovery | 0.11 [0.05, 0.16] | 0.17 | -0.24 [-0.29, -0.18] | 0.45 [0.4, 0.5] |
| heartmath | LnRMSSD | standing | 0.32 [0.21, 0.42] | 0.33 | -0.33 [-0.44, -0.23] | 0.97 [0.87, 1.07] |
| heartmath | LnRMSSD | breathing | -0.01 [-0.05, 0.02] | 0.11 | -0.23 [-0.26, -0.19] | 0.2 [0.17, 0.24] |
| heartmath | LnRMSSD | neurotask | 0.08 [0.01, 0.14] | 0.2 | -0.32 [-0.39, -0.26] | 0.48 [0.41, 0.54] |
| heartmath | LnRMSSD | walking | 0.5 [0.36, 0.64] | 0.45 | -0.38 [-0.52, -0.24] | 1.38 [1.24, 1.52] |
| heartmath | LnRMSSD | biking | 2.14 [1.81, 2.47] | 1.03 | 0.11 [-0.21, 0.43] | 4.16 [3.84, 4.48] |
| heartmath | LnHF | sitting | 0.18 [0.08, 0.27] | 0.3 | -0.41 [-0.5, -0.32] | 0.76 [0.67, 0.85] |
| heartmath | LnHF | arithmetic | 0.25 [0.13, 0.38] | 0.39 | -0.51 [-0.63, -0.39] | 1.02 [0.9, 1.14] |
| heartmath | LnHF | recovery | 0.09 [0.04, 0.14] | 0.15 | -0.22 [-0.26, -0.17] | 0.39 [0.34, 0.44] |
| heartmath | LnHF | standing | 0.28 [0.16, 0.41] | 0.39 | -0.47 [-0.59, -0.35] | 1.04 [0.92, 1.16] |
| heartmath | LnHF | breathing | 0.09 [0.02, 0.15] | 0.2 | -0.31 [-0.37, -0.25] | 0.49 [0.42, 0.55] |
| heartmath | LnHF | neurotask | 0.17 [0.06, 0.27] | 0.33 | -0.48 [-0.58, -0.37] | 0.81 [0.71, 0.91] |
| heartmath | LnHF | walking | 0.45 [0.29, 0.61] | 0.51 | -0.54 [-0.7, -0.38] | 1.45 [1.29, 1.6] |
| heartmath | LnHF | biking | 3.92 [2.98, 4.86] | 2.94 | -1.83 [-2.74, -0.92] | 9.68 [8.77, 10.59] |
| heartmath | Mean HR | sitting | 1.85 [1.71, 2.0] | 0.45 | 0.97 [0.83, 1.11] | 2.74 [2.6, 2.87] |
| heartmath | Mean HR | arithmetic | 2.06 [1.81, 2.32] | 0.79 | 0.51 [0.26, 0.76] | 3.62 [3.37, 3.86] |
| heartmath | Mean HR | recovery | 1.73 [1.61, 1.86] | 0.4 | 0.96 [0.83, 1.08] | 2.51 [2.39, 2.63] |
| heartmath | Mean HR | standing | 1.97 [1.89, 2.06] | 0.27 | 1.44 [1.36, 1.53] | 2.5 [2.42, 2.58] |
| heartmath | Mean HR | breathing | 1.91 [1.59, 2.22] | 0.98 | -0.02 [-0.33, 0.28] | 3.84 [3.53, 4.14] |
| heartmath | Mean HR | neurotask | 1.85 [1.7, 2.0] | 0.48 | 0.92 [0.77, 1.06] | 2.78 [2.64, 2.93] |
| heartmath | Mean HR | walking | 2.15 [2.0, 2.31] | 0.49 | 1.2 [1.04, 1.35] | 3.11 [2.96, 3.26] |
| heartmath | Mean HR | biking | -10.03 [-16.38, -3.68] | 19.86 | -48.96 [-55.12, -42.81] | 28.9 [22.75, 35.06] |
| empatica | LnRMSSD | sitting | 0.33 [0.2, 0.46] | 0.37 | -0.4 [-0.52, -0.27] | 1.06 [0.94, 1.19] |
| empatica | LnRMSSD | arithmetic | 0.92 [0.74, 1.11] | 0.53 | -0.11 [-0.29, 0.06] | 1.96 [1.78, 2.14] |
| empatica | LnRMSSD | recovery | 0.45 [0.32, 0.58] | 0.38 | -0.29 [-0.41, -0.16] | 1.19 [1.06, 1.32] |
| empatica | LnRMSSD | standing | 0.76 [0.57, 0.94] | 0.53 | -0.28 [-0.46, -0.1] | 1.79 [1.62, 1.97] |
| empatica | LnRMSSD | breathing | 0.18 [0.09, 0.27] | 0.26 | -0.32 [-0.4, -0.23] | 0.68 [0.6, 0.77] |
| empatica | LnRMSSD | neurotask | 0.49 [0.34, 0.64] | 0.42 | -0.33 [-0.47, -0.19] | 1.31 [1.17, 1.45] |
| empatica | LnRMSSD | walking | 1.45 [1.29, 1.61] | 0.44 | 0.59 [0.44, 0.74] | 2.32 [2.17, 2.47] |
| empatica | LnRMSSD | biking | 2.79 [2.55, 3.04] | 0.67 | 1.48 [1.24, 1.71] | 4.11 [3.87, 4.34] |
| empatica | LnHF | sitting | 0.33 [0.12, 0.54] | 0.61 | -0.86 [-1.06, -0.66] | 1.52 [1.32, 1.72] |
| empatica | LnHF | arithmetic | 1.44 [1.06, 1.83] | 1.11 | -0.74 [-1.11, -0.36] | 3.62 [3.25, 4.0] |
| empatica | LnHF | recovery | 0.53 [0.31, 0.75] | 0.62 | -0.69 [-0.9, -0.48] | 1.76 [1.55, 1.97] |
| empatica | LnHF | standing | 1.11 [0.71, 1.51] | 1.14 | -1.13 [-1.51, -0.75] | 3.36 [2.97, 3.74] |
| empatica | LnHF | breathing | 0.29 [0.12, 0.47] | 0.52 | -0.71 [-0.89, -0.54] | 1.3 [1.13, 1.48] |
| empatica | LnHF | neurotask | 0.81 [0.53, 1.08] | 0.78 | -0.72 [-0.98, -0.46] | 2.33 [2.07, 2.59] |
| empatica | LnHF | walking | 3.14 [2.81, 3.46] | 0.89 | 1.39 [1.08, 1.7] | 4.89 [4.58, 5.2] |
| empatica | LnHF | biking | 6.01 [5.47, 6.55] | 1.47 | 3.13 [2.61, 3.65] | 8.89 [8.37, 9.41] |
| empatica | Mean HR | sitting | -0.15 [-0.66, 0.36] | 1.46 | -3.01 [-3.49, -2.52] | 2.71 [2.22, 3.2] |
| empatica | Mean HR | arithmetic | -5.79 [-8.91, -2.66] | 8.95 | -23.33 [-26.34, -20.33] | 11.76 [8.75, 14.77] |
| empatica | Mean HR | recovery | -0.35 [-1.01, 0.31] | 1.89 | -4.05 [-4.69, -3.42] | 3.36 [2.73, 4.0] |
| empatica | Mean HR | standing | -0.63 [-1.87, 0.61] | 3.56 | -7.59 [-8.79, -6.4] | 6.34 [5.15, 7.54] |
| empatica | Mean HR | breathing | 0.06 [-0.32, 0.44] | 1.09 | -2.07 [-2.44, -1.7] | 2.2 [1.83, 2.56] |
| empatica | Mean HR | neurotask | -0.44 [-1.0, 0.12] | 1.6 | -3.58 [-4.12, -3.04] | 2.7 [2.16, 3.24] |
| empatica | Mean HR | walking | -5.38 [-8.95, -1.81] | 9.9 | -24.78 [-28.21, -21.35] | 14.02 [10.59, 17.45] |
| empatica | Mean HR | biking | -38.0 [-45.98, -30.02] | 21.76 | -80.65 [-88.32, -72.99] | 4.65 [-3.01, 12.31] |

Supplementary Table 5 - Summary of Bland-Altman Analysis Results. This table presents the bias and limits of agreement (LoAs) for various devices across different conditions and features, compared against the criterion device (VU-AMS). Each row includes the calculated bias and LoAs, accompanied by their respective 95% Confidence Intervals (CIs) in brackets. LnRMSSD: Log-transformed root mean square of successive differences; LnHF: log-transformed of high frequency component; Mean HR: mean heart rate.


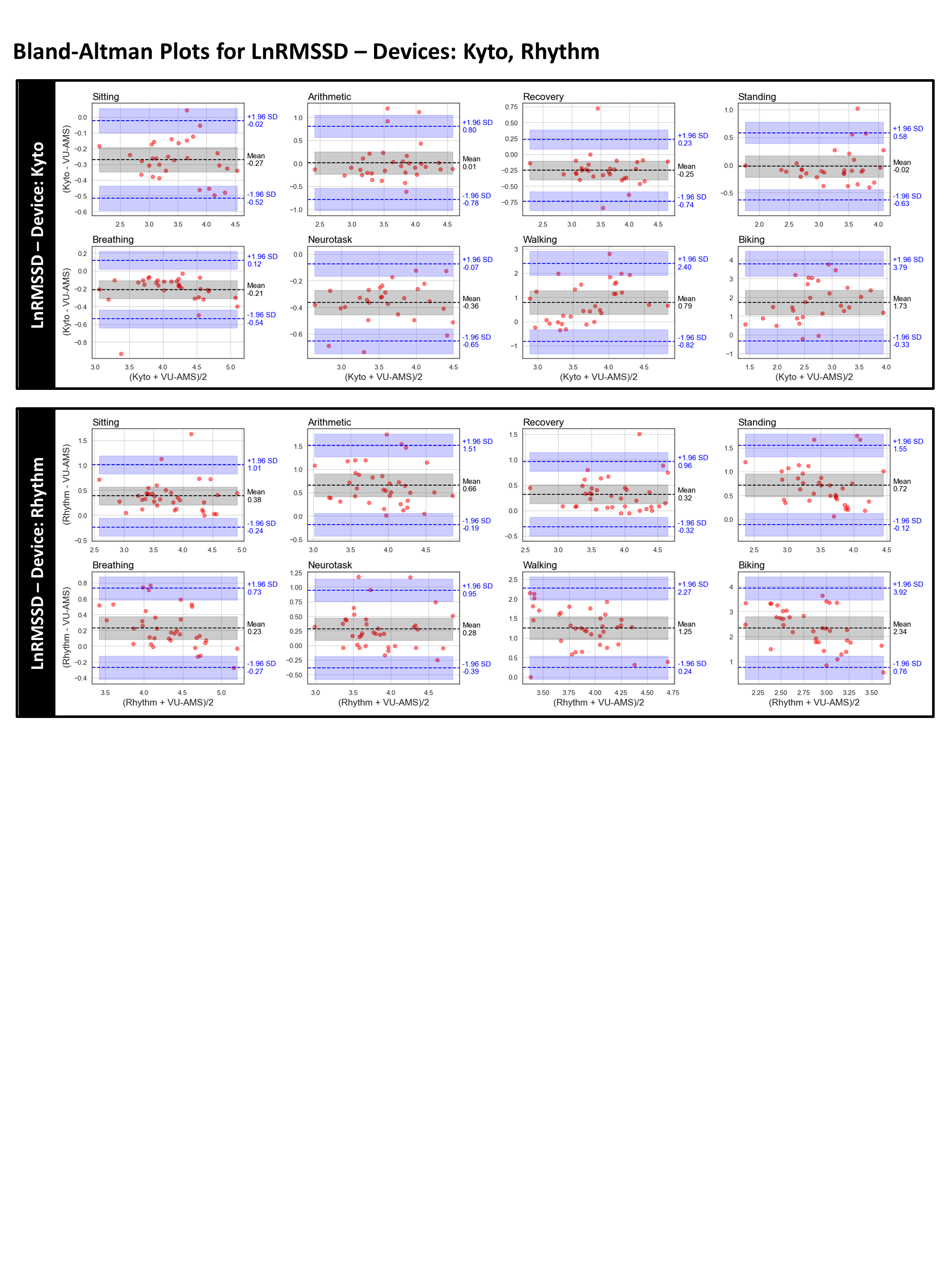


Supplementary Figure 7 - Bland-Altman Plots for LnRMMSD for Kyto and Rhythm devices: Each subplot represents the comparison between a measurement device and the criterion device for LnRMSSD (log-transformed root mean square of successive differences) under a particular condition. The central dashed line in each plot indicates the mean difference (bias) between the device and the criterion, the upper and lower dashed lines represent the limits of agreement. Data points (in red) show the differences between the criterion and device (y-axis) plotted against the mean of the device and criterion measurements (x-axis). The shaded areas around the mean difference and limits of agreement lines indicates the confidence intervals (95%).


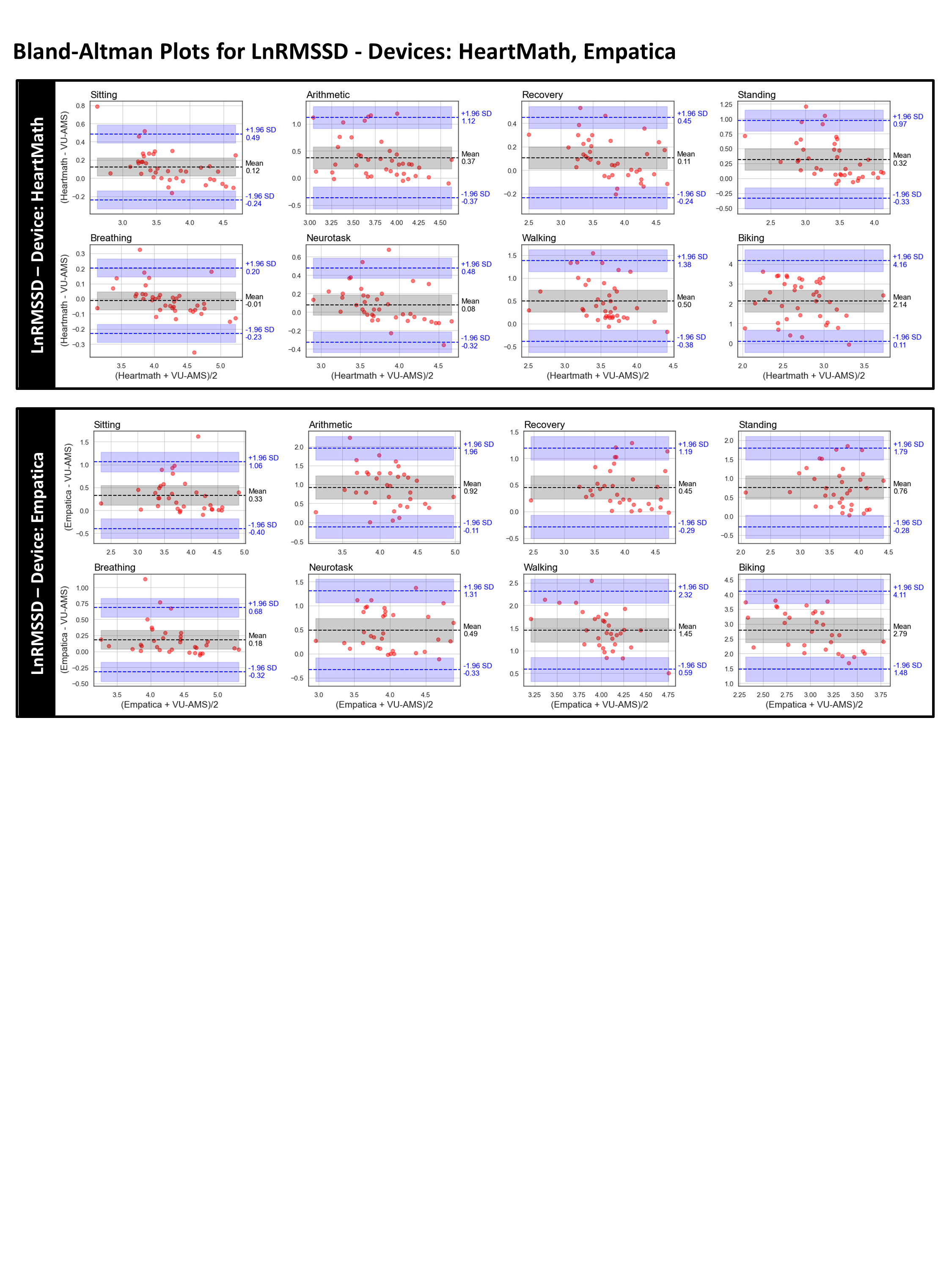


Supplementary Figure 8 - Bland-Altman Plots for LnRMMSD for Heartmath and Empatica devices: Each subplot represents the comparison between a measurement device and the criterion device for LnRMSSD (log-transformed root mean square of successive differences) under a particular condition. The central dashed line in each plot indicates the mean difference (bias) between the device and the criterion, the upper and lower dashed lines represent the limits of agreement. Data points (in red) show the differences between the criterion and device (y-axis) plotted against the mean of the device and criterion measurements (x-axis). The shaded areas around the mean difference and limits of agreement lines indicates the confidence intervals (95%).


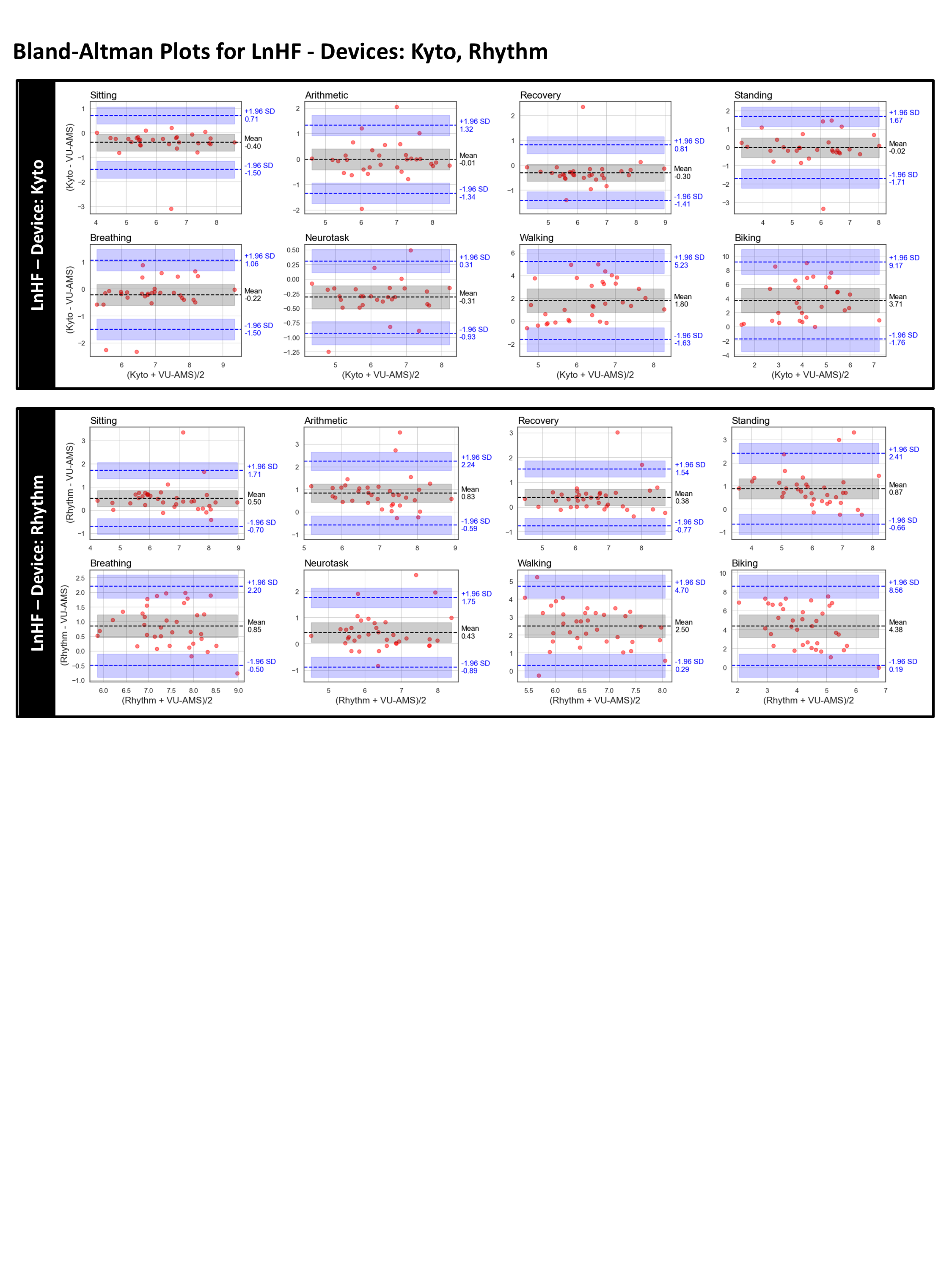


Supplementary Figure 9 - Bland-Altman Plots for LnHF for Kyto and Rhythm devices: Each subplot represents the comparison between a measurement device and the criterion device for LnHF (log-transformed high frequency component) under a particular condition. The central dashed line in each plot indicates the mean difference (bias) between the device and the criterion, the upper and lower dashed lines represent the limits of agreement. Data points (in red) show the differences between the criterion and device (y-axis) plotted against the mean of the device and criterion measurements (x-axis). The shaded areas around the mean difference and limits of agreement lines indicates the confidence intervals (95%).


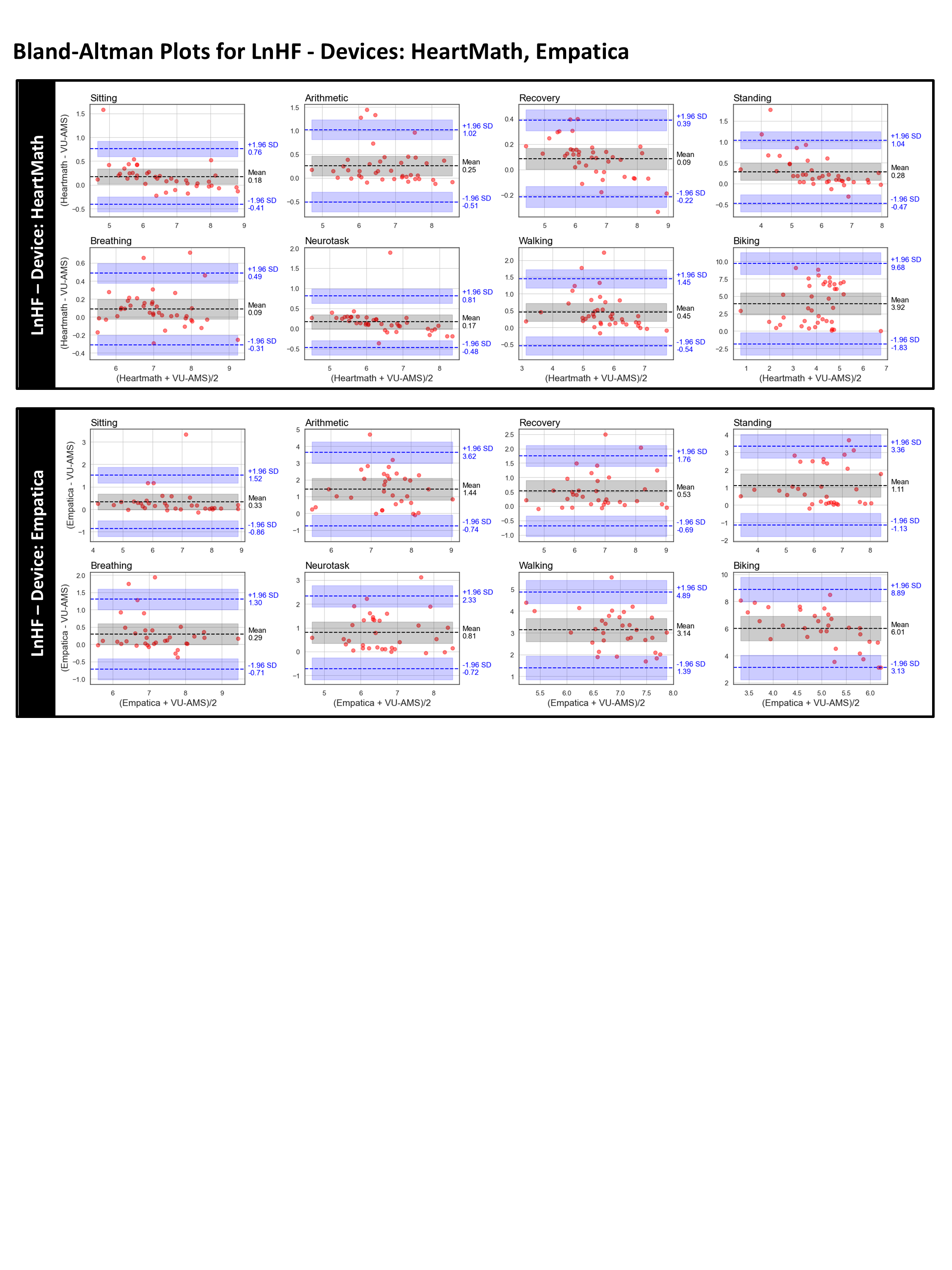


Supplementary Figure 10 - Bland-Altman Plots for LnHF for HeartMath and Empatica devices: Each subplot represents the comparison between a measurement device and the criterion device for LnHF (log-transformed high frequency component) under a particular condition. The central dashed line in each plot indicates the mean difference (bias) between the device and the criterion, the upper and lower dashed lines represent the limits of agreement. Data points (in red) show the differences between the criterion and device (y-axis) plotted against the mean of the device and criterion measurements (x-axis). The shaded areas around the mean difference and limits of agreement lines indicates the confidence intervals (95%).


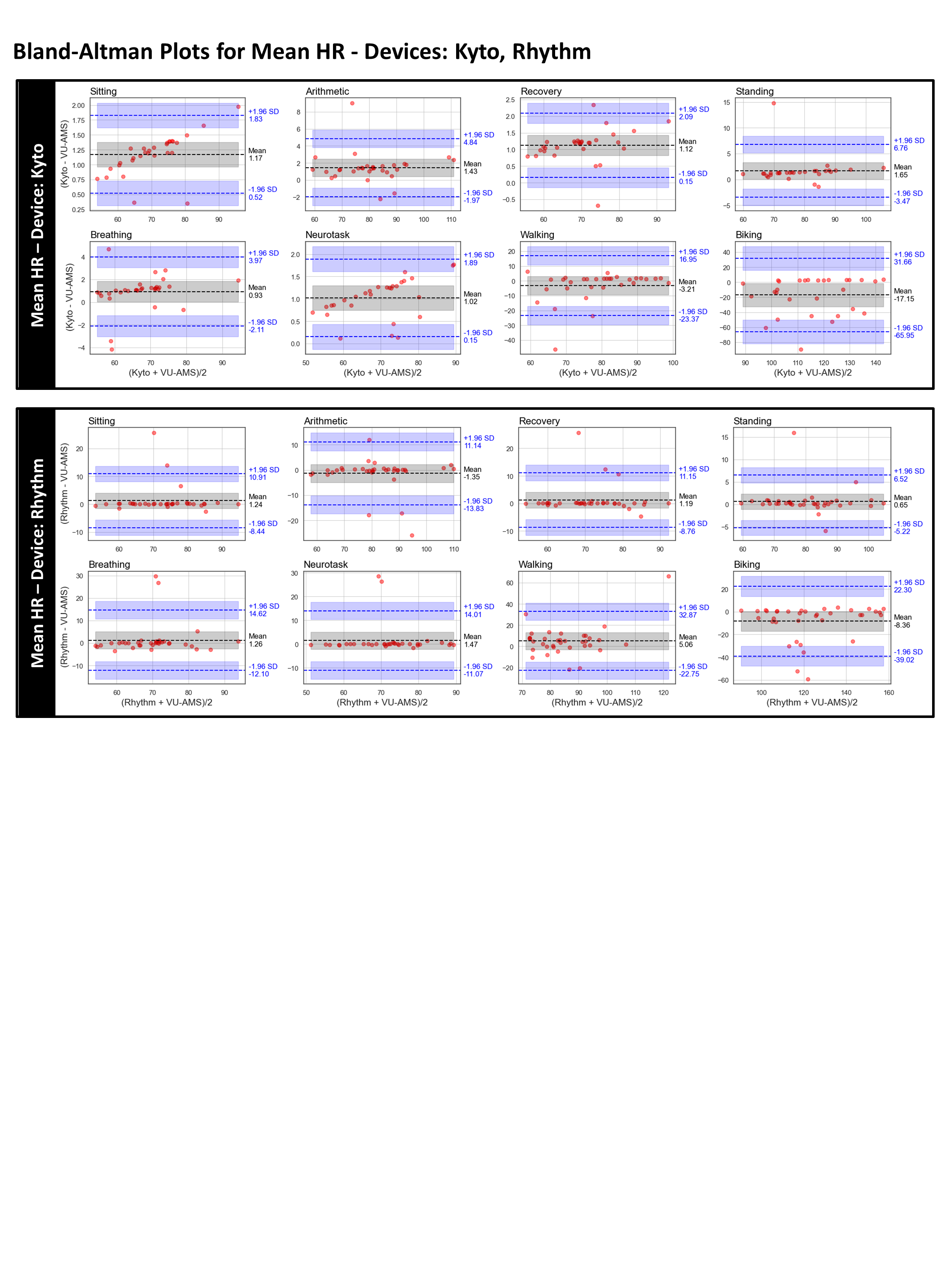


Supplementary Figure 11 - Bland-Altman Plots for Mean HR for Kyto and Rhythm devices: Each subplot represents the comparison between a measurement device and the criterion device for Mean HR (mean heart rate) under a particular condition. The central dashed line in each plot indicates the mean difference (bias) between the device and the criterion, the upper and lower dashed lines represent the limits of agreement. Data points (in red) show the differences between the criterion and device (y-axis) plotted against the mean of the device and criterion measurements (x-axis). The shaded areas around the mean difference and limits of agreement lines indicates the confidence intervals (95%).


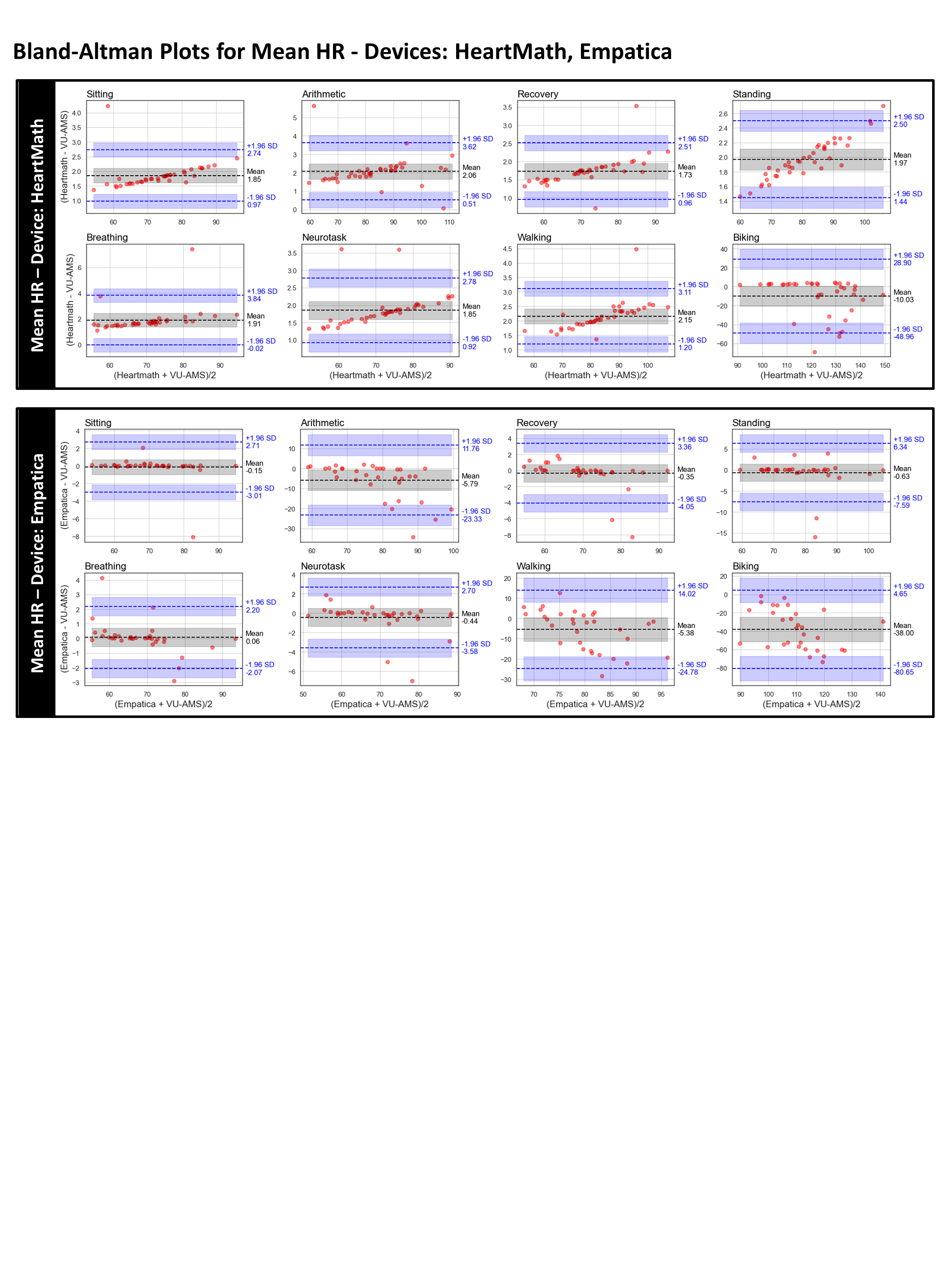


Supplementary Figure 12 - Bland-Altman Plots for Mean HR for HeartMath and Empatica devices: Each subplot represents the comparison between a measurement device and the criterion device for Mean HR (mean heart rate) under a particular condition. The central dashed line in each plot indicates the mean difference (bias) between the device and the criterion, the upper and lower dashed lines represent the limits of agreement. Data points (in red) show the differences between the criterion and device (y-axis) plotted against the mean of the device and criterion measurements (x-axis). The shaded areas around the mean difference and limits of agreement lines indicates the confidence intervals (95%).

# Additional

## Raw ECG Versus Raw PPG

To further illustrate the limitations of PPG signals, we provide a 30-second example from a single participant at the beginning of the sitting and biking conditions (P30, whose interbeat intervals are shown in Figure 3 of the main paper). During the sitting condition, the PPG waveform appears clean, with visually clear peaks. We then used Neurokit2 (Makowski et al., 2021) to clean and detect peaks in this signal. The results are displayed in the plot below, where the detected IBIs from the VU-AMS are compared against the PPG peaks detected using Neurokit2 and the systolic peaks identified by Empatica’s original algorithm (used in this study). The three sets of data closely align. However, during the biking condition, while the raw ECG signal remains clean, the raw PPG signal becomes distorted, making peak detection challenging. As a result, both the Neurokit algorithm and the device’s original algorithm do not compare well against the reference VU-AMS IBIs, which are derived from the ECG.


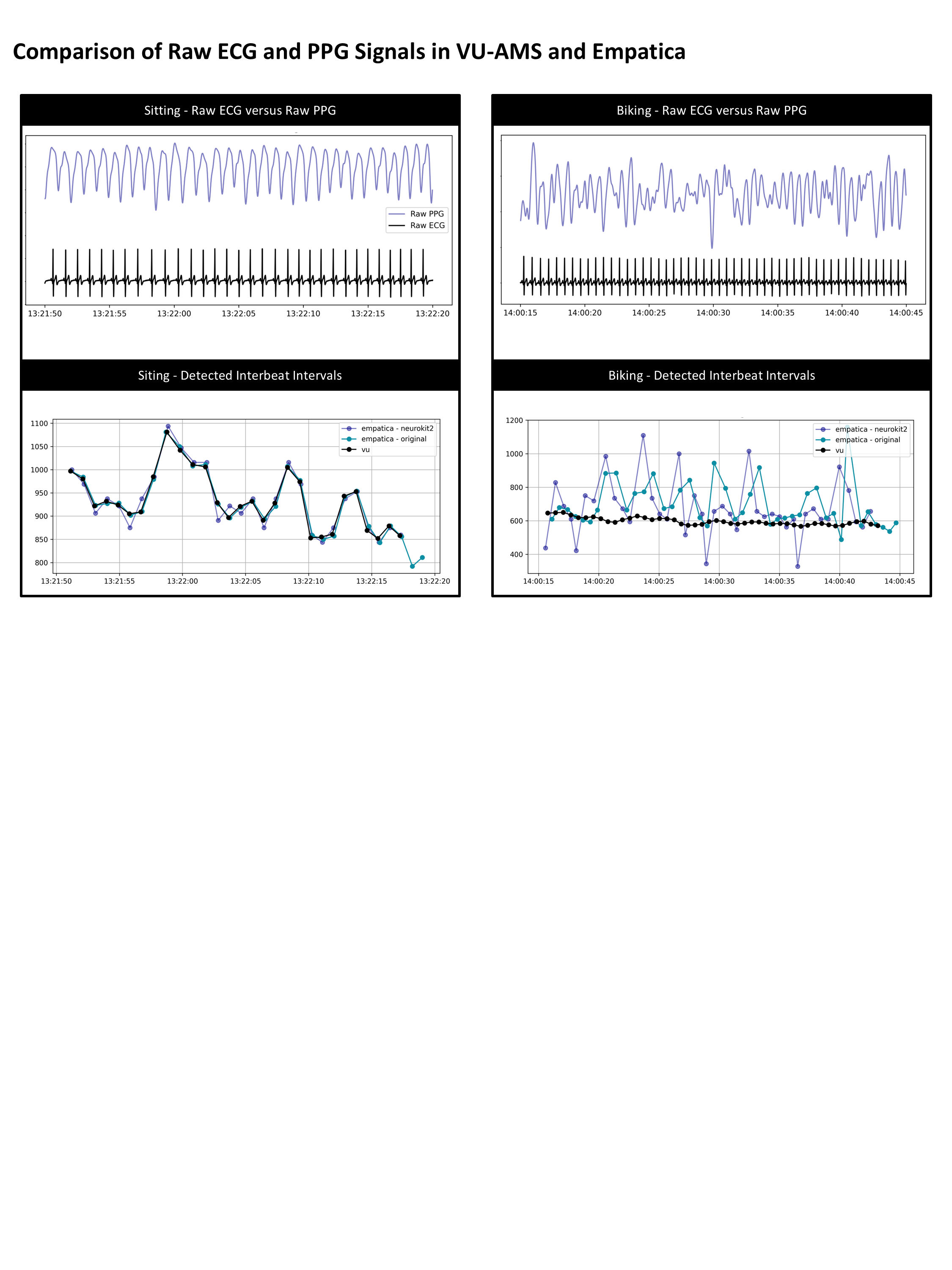


Supplementary Figure 13 - Comparison of Raw ECG and PPG Signals in VU-AMS and Empatica Devices (30 seconds, Participant P30): Each subplot represents a condition (sitting and biking), comparing raw ECG and PPG signals, as well as the detected interbeat intervals (IBIs) from the VU-AMS and Empatica devices for a 30-second segment from one representative participant (P30). The upper plots show raw ECG and PPG signals, while the lower plots display the detected IBIs from VU-AMS, Neurokit2-detected PPG peaks, and Empatica’s original algorithm. Differences in PPG signal quality between sitting and biking conditions are highlighted, showing alignment during sitting and discrepancies during biking.
